# COCOA: A Framework for Fine-scale Mapping of Cell-type-specific Chromatin Compartments Using Epigenomic Information

**DOI:** 10.1093/gpbjnl/qzae091

**Published:** 2024-12-26

**Authors:** Kai Li, Ping Zhang, Jinsheng Xu, Zi Wen, Junying Zhang, Zhike Zi, Li Li

**Affiliations:** Hubei Key Laboratory of Agricultural Bioinformatics, College of Informatics, Huazhong Agricultural University, Wuhan 430070, China; Hubei Key Laboratory of Agricultural Bioinformatics, College of Informatics, Huazhong Agricultural University, Wuhan 430070, China; Hubei Key Laboratory of Agricultural Bioinformatics, College of Informatics, Huazhong Agricultural University, Wuhan 430070, China; Hubei Key Laboratory of Agricultural Bioinformatics, College of Informatics, Huazhong Agricultural University, Wuhan 430070, China; Hubei Key Laboratory of Agricultural Bioinformatics, College of Informatics, Huazhong Agricultural University, Wuhan 430070, China; Shenzhen Key Laboratory of Synthetic Genomics, Guangdong Provincial Key Laboratory of Synthetic Genomics, Key Laboratory of Quantitative Synthetic Biology, Shenzhen Institute of Synthetic Biology, Shenzhen Institute of Advanced Technology, Chinese Academy of Sciences, Shenzhen 518055, China; Hubei Key Laboratory of Agricultural Bioinformatics, College of Informatics, Huazhong Agricultural University, Wuhan 430070, China; Hubei Hongshan Laboratory, Wuhan 430070, China

**Keywords:** Three-dimensional genome, Deep learning, Chromatin compartment, Histone modification, Cell-type specificity

## Abstract

Chromatin compartmentalization and epigenomic modifications play crucial roles in cell differentiation and disease development. However, precise mapping of chromatin compartment patterns requires Hi-C or Micro-C data at high sequencing depth. Exploring the systematic relationship between epigenomic modifications and compartment patterns remains challenging. To address these issues, we present COCOA, a deep neural network framework using convolution and attention mechanisms to infer fine-scale chromatin compartment patterns from six histone modification signals. COCOA extracts 1D track features through bidirectional feature reconstruction after resolution-specific binning of epigenomic signals. These track features are then cross-fused with contact features using an attention mechanism and transformed into chromatin compartment patterns through residual feature reduction. COCOA demonstrates accurate inference of chromatin compartmentalization at a fine-scale resolution and exhibits stable performance on test sets. Additionally, we explored the impact of histone modifications on chromatin compartmentalization prediction through *in silico* epigenomic perturbation experiments. Unlike obscure compartments observed in high-depth experimental data at 1-kb resolution, COCOA generates clear and detailed compartment patterns, highlighting its superior performance. Finally, we demonstrate that COCOA enables cell-type-specific prediction of unrevealed chromatin compartment patterns in various biological processes, making it an effective tool for gaining insights into chromatin compartmentalization from epigenomics in diverse biological scenarios. The COCOA Python code is publicly available at https://github.com/onlybugs/COCOA and https://ngdc.cncb.ac.cn/biocode/tools/BT007498.

## Introduction

The three-dimensional (3D) architecture of chromatin is essential for gene expression regulation during cell differentiation and disease development [1,2]. Recent advances in next-generation sequencing have led to the development of several chromosome conformation capture techniques, such as Hi-C, Micro-C, and Pore-C [3–5], enabling the exploration of multiscale chromatin structural elements including chromatin compartments [3,6], topological associating domains (TADs) [7,8], loops [6], stripes [9], and microcompartments [10]. These techniques have revealed that the chromatin can be segregated into A and B compartments [3,11]. The A compartments are generally active chromatin, whereas the B compartments are mostly transcriptionally repressive. These chromatin compartments are closely related to the mechanisms underlying various key biological processes [12,13].

To identify chromatin compartments, sequencing data are usually processed into contact maps, and distance effects are eliminated using normalization methods. The normalized contact map is then used to calculate the correlation matrix (CM), which is subjected to principal component analysis (PCA). The sign of the first principal component (PC1) corresponds to the compartment state [3]. Most analyses related to chromatin compartments rely on CM and PC1 [14–16]. While the CM is commonly available and of high quality at mega-base scale, it becomes noisy at resolutions finer than 25 kb, failing to show clear plaid patterns due to its sparseness. Recent studies have suggested the associations of the fine-scale-resolution chromatin compartments with other structural elements [17,18], histone modifications, and chromatin accessibility [19]. However, the available chromatin compartment data do not match the scale of the epigenomic data, making the connection between epigenomics and chromatin compartmentalization a challenge. Furthermore, due to technical limitations and sequencing costs [20], experimentally mapping high-resolution chromatin compartments is both expensive and labor-intensive. Therefore, there is an unmet need for the development of a computational method to obtain the fine-scale CM across multiple cell lines.

In the past decade, deep learning [21] has emerged as a widely used tool in computational 3D genomics. These applications include various tasks such as TAD boundary recognition [22,23], chromatin loop detection [24,25], chromatin interaction data enhancement [26,27], interaction matrix generation [28–30], and single-cell Hi-C imputation [31,32]. While several methods explore contact map generation and enhancement, they lack cell-type specificity. For example, HiC-Reg [33] uses fourteen epigenomic signals from five cell lines to predict short-range chromatin interactions using random forests. Akita [29] and Orca [34] adopt convolutional neural networks to predict contact maps from DNA sequences. However, these methods are not capable of directly inferring contact maps across different cell types. Recently, two proposed methods, C.Origami [35] and Epiphany [30], address this limitation by utilizing histone modifications and chromatin accessibility data. C.Origami predicts short-range interactions by integrating CCCTC-binding factor (CTCF), chromatin accessibility, and DNA sequence information through a neural network containing the attention and convolutional modules. Epiphany uses multiple epigenomic signals to generate short-range chromatin contact maps. However, these methods have their own limitations in terms of chromatin compartmentation and method generalization. Firstly, these existing methods concentrate on the prediction of short-range interactions (TADs and loops) while ignoring long-range interactions (compartments). Additionally, the relationship between compartmentalization and histone modifications is still unresolved. Furthermore, these models require inputs in fixed bin sizes, limiting scalability and preventing across-resolution predictions.

To resolve these limitations, we introduce COCOA, a method that predicts the cell-type-specific CM using six types of accessible epigenomic modification signals. COCOA adopts bidirectional feature reconstruction and cross-attention fusion for bidirectional reconstruction and fusion of epigenomic data. Subsequently, residual feature reduction is applied to map the fused results into CM. COCOA is specifically designed to generate chromatin compartmentalization, and the predicted CM can be directly used to determine compartment statuses. We evaluated the performance of COCOA using multiple metrics, including mean square error (MSE), mean absolute error (MAE), GenomeDISCO score, Pearson correlation coefficient (PCC), structure similarity (SSIM) index, and peak signal to noise ratio (PSNR). The results demonstrate that COCOA accurately generates significant and biologically meaningful CMs. Furthermore, we conducted *in silico* perturbation experiments to investigate the influence of histone modifications on compartment prediction. Additionally, we tested the generalization performance of COCOA by making model predictions with resolution-specific and cell-type-specific data. The results show that COCOA enables robust performance at various resolutions across diverse cell lines, providing insights into the patterns of chromatin compartments in immune and disease tissues.

## Method

### Hi-C and Micro-C data sources and preprocessing

We collected publicly available processed Hi-C and Micro-C data of different cell lines from the 4DN database [36]. Intra-chromosomal contact maps were computed from these data for model training and testing (Table S1). Depending on the specific task, the intra-chromosomal contact maps were computed at different resolutions using the cooler package [37]. To eliminate the distance effect in the contact maps, we applied the observed-expected (OE) normalization method [3]. Finally, these normalized contact maps were converted into CMs, which clearly depict the plaid pattern of chromatin compartmentalization.

### ChIP-seq data sources and preprocessing

**Figure 1:**
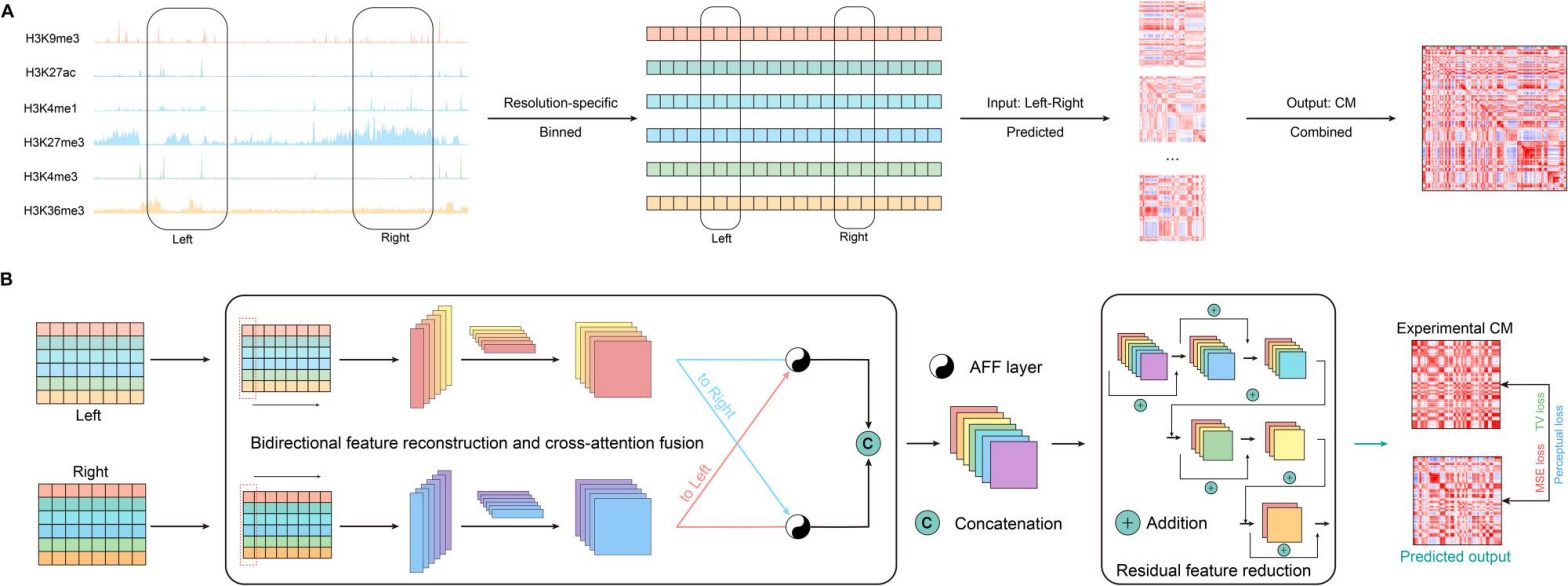
COCOA pipeline and architecture **A**. COCOA pipeline: the integration of six accessible epigenomic signals by resolution-specific binning serves as inputs to predict the CM. **B**. COCOA architecture: COCOA extracts 1D track features from each input (the bidirectional feature reconstruction module) and then combines these features with spatial contact features (the cross-attention fusion module). The contact features are further processed by the residual feature reduction module to obtain the final prediction result. Parameters are updated using a backpropagation algorithm with mixed loss functions. Refer to Method section for detailed information. CM, correlation matrix; AFF, attention feature fusion.

Histone modification signals (H3K9me3, H3K27ac, H3K4me1, H3K27me3, H3K4me3, and H3K36me3) from the ChIP-seq [38] data for all cells were retrieved from the ENCODE project [39] (Table S2). The ChIP-seq data were binned to specific resolutions using the pyBigWig package (**Figure 1**A). After binning, a log(x+1) transformation and min-max normalization were performed on the data. Finally, the processed data were combined into an epigenomic matrix (EM).

### Dividing matrices

The preprocessing step generates two matrices: a symmetric CM with dimensions *n*×*n* ($CM_{n\times n}$) and an EM with dimensions *m*×*n* ($EM_{m\times n}$). Each $CM_{ij}$ in the $CM_{n\times n}$ represents the correlation strength between genomic segments i and j. Values greater than 1 indicate that the two genomic segments have the same interaction mode, while values less than 1 indicate the opposite interaction mode, providing information about the status of chromatin compartments. Each $EM_{ij}$ in the $EM_{m\times n}$ represents the signal strength of genomic segment j on epigenomic track i.

To better preserve the plaid pattern of chromatin compartmentalization and adapt to the inputs of the neural network, we implemented the following processing scheme. First, the $CM_{n\times n}$ was divided into sub-matrices of k×k size ($SCM_{k\times k}$), and the $EM_{m\times n}$ was divided into two sub-matrices of m×k size ($SLEM_{m\times k}$ and $SREM_{m\times k}$). We started at the diagonal position in the top-left corner of $CM_{n\times n}$ and moved horizontally, dividing it into $SCM_{k\times k}$. Simultaneously, we divided the two corresponding groups of genomic loci from EM into $SLEM_{m\times k}$ and $SREM_{m\times k}$. After completing the horizontal division, we moved the current position diagonally by *k* positions. This process was repeated until the entire $CM_{n\times n}$ could no longer be divided. Due to computational resource constraints, we sampled the $SCM_{k\times k}$ in groups to minimize the size of the training datasets ($SLEM_{m\times k}$ and $SREM_{m\times k}$ were synchronized to minimize). Finally, these data were saved separately for further modeling.

### Combining predicted sub-matrices

The COCOA model takes the $SLEM_{m\times k}$ and $SREM_{m\times k}$ for each chromosome division as inputs. It then outputs a series of predicted correlation sub-matrices. These sub-matrices sequentially cover a square matrix ($PCM_{n\times n}$) with the same number of columns as the $EM_{m\times n}$. The specific coordinates for covering each predicted correlation sub-matrix are determined by the corresponding inputs ($SLEM_{m\times k}$ and $SREM_{m\times k}$). Finally, the complete $PCM_{n\times n}$ is generated and saved for further biological analyses.

### COCOA architecture

The COCOA model consists of three main components: bidirectional feature reconstruction, cross-attention fusion, and residual feature reduction (Figure 1B), which are described in the following sections.

### Bidirectional feature reconstruction

The bidirectional feature reconstruction module consists of two matrix reconstruction (MR) layers. The construction of these MR layers is inspired by our previous work on chromatin interaction data enhancement [40]. Each MR layer consists of two parts: an aggregation convolution layer with a filter size of N×1 and a linear reconstruction layer. The output of each MR layer is computed by Equations 1 and 2:

(1) $$\begin{array}{c} v\left(SEM\right)=Tanh\left(\left(SEM\;⊗\;K_{N\times 1}\right)\times W_{i}\right) \end{array}$$

 

(2) $$\begin{array}{c} MR\left(SEM\right)=v\left(SEM\right)\cdot \;{v\left(SEM\right)}^{T}\times W_{j} \end{array}$$

where ⊗ denotes the convolution operation, $K_{N\times 1}$ represents convolution kernel (N×1), Tanh is the activation function [41], × denotes Hadamard product, and · denotes dot product. SEM represents $SLEM_{m\times k}$ or $SREM_{m\times k}$ generated through preprocessing. $v^{T}$ represents the transposition of the vector v. $W_{i}$ and $W_{j}$ are learnable weight matrices, respectively. The MR layer aggregates multiple ChIP-seq track signals from different genomic loci into a 1D vector. This vector is in turn reconstructed into a low-ranking epigenomic track feature using learnable weight matrices. In summary, this module obtains bidirectional epigenomic track features by reconstructing the $SLEM_{m\times k}$ and $SREM_{m\times k}$.

### Cross-attention fusion

Next, the COCOA model employs the cross-attention fusion module to fuse bidirectional epigenomic track features. This module mainly contains two attention feature fusion (AFF) layers [42]. Each AFF layer has three parts: global feature extraction, local feature extraction, and attention fusion. The results of cross-attention fusion are defined by Equation 3:

(3) $$\begin{array}{c} CAF\left(P,Q\right)=concat(AFF_{1}\left(P,Q\right),AFF_{2}(Q,P)) \end{array}$$

where *P* and *Q* represent bidirectional epigenomic track features, respectively, concat denotes stacking two outputs in the same dimension, and AFF refers to an attention-based uniform and general neural network layer for feature fusion proposed by Dai et al. [42]. The cross-attention fusion module transforms epigenomic track features from the other direction into potential attention weights to reinforce the epigenomic track features in the current direction. By interleaving attention fusion and concatenation, a set of fused contact feature maps is obtained as inputs for the next module.

### Residual feature reduction

The residual feature reduction module consists of a series of residual blocks, each containing several residual layers. Following the approach described in previous work [43], each residual layer is composed of convolutional layers with different convolution kernels, batch normalization (BN) layers [44], and activation functions. The computation of each layer is defined by Equations 4 and 5:

(4) $$\begin{array}{c} F\left(X\right)=Tanh\left(BN_{1}\left(X⊗K_{n\times n}\right)\right) \end{array}$$

 

(5) $$\begin{array}{c} Res\left(X\right)=Tanh\left(BN_{2}\left(F\left(X\right)⊗K_{m\times m}\right)+X\right) \end{array}$$

where K denotes the convolution kernels of different sizes, Tanh is the activation function, and BN represents the BN layer. X represents the fused contact feature maps for the first layer, and the output of the current layer serves as the input for the next layer. The residual feature reduction module decreases the channels of the contact features from the previous module, level by level. Throughout this process, the residual layer continuously filters to retain important information from the previous layer, aggregating it with the output of the current layer. Finally, the predicted correlation sub-matrix is obtained from the last layer of the residual feature reduction module.

### Loss function

The COCOA model can be viewed as a function F with a parameter set θ, which maps each group input $SLEM_{i,m\times k}$ and $SREM_{i,m\times k}$ to the predicted correlation sub-matrix $PSCM_{i,k\times k}$ [*i.e.*, $PSCM_{i,k\times k}=F(SLEM_{i,m\times k},SREM_{i,m\times k}\;:\;\theta )$]. The training objective is to find a set of ${\;\theta }^{*}$ to enable $PSCM_{i,k\times k}$ similar to the ground truth $SCM_{i,k\times k}$. Therefore, COCOA initially uses the MSE loss to minimize the pairwise error of the genomic range k×k between PSCM and SCM. This loss can be described as Equation 6:

(6) $$\begin{array}{c} L_{MSE}\left(PSCM_{i,k\times k},SCM_{i,k\times k}\right)=\frac{1}{m}\sum_{i=1}^{m}{\left(PSCM_{i,k\times k}-SCM_{i,k\times k}\right)}^{2} \end{array}$$

Subsequently, COCOA incorporates a perceptual loss based on the Visual Geometry Group (VGG) network [45] to restore structural information of the CM. Furthermore, the total variation (TV) loss [46] is added, which effectively smooths noise in computer vision, as a regularization term to suppress the noise of the $PSCM_{k\times k}$. These losses are described as Equations 7 and 8:

(7) $$H_{VGG}\left(PSCM_{i,k\times k},SCM_{i,k\times k}\right)=\frac{1}{N}\sum_{k=1}^{N}{\left(VGG\left(PSCM_{i,k\times k}\right)-VGG\left(SCM_{i,k\times k}\right)\right)}^{2}$$

 

(8) $$F_{TV}\left(PSCM_{k\times k}\right)=\sum_{i,j}{\left(PSCM_{i,j-1}-PSCM_{i,j}\right)}^{2}+\sum_{i,j}{\left(PSCM_{i+1,j}-PSCM_{i,j}\right)}^{2}$$

Finally, the training objective is represented by Equation 9:

(9) $$\theta^{*}=argmin_{\theta }[L_{MSE}\left(PSCM_{i,k\times k},SCM_{i,k\times k}\right)+{\alpha *F}_{TV}\left(PSCM_{i,k\times k}\right)+\beta *H_{VGG}\left(PSCM_{i,k\times k},SCM_{i,k\times k}\right)]$$

where α and β are scaling weights that range from 0 to 1.

### COCOA training and hyperparameter exploration

Before model training, we preprocessed each chromosome of the HFFc6 Micro-C data [47] and corresponding ChIP-seq data. Chromosomes 1, 3, 5, 7, 9, 11, 13, 15, 17, and 19 were used as training sets, while chromosomes 18, 20, 21, and 22 were utilized for hyperparameter tuning. The remaining chromosomes were allocated for performance evaluation.

The COCOA model was implemented in Python 3.7 with PyTorch1.12 [48]. We trained the model with a batch size of 16 for 120 epochs, using the Adam optimizer [49] with an initial learning rate of 5E–4 ($lr_{init}=5E–4$). All the training and testing processes were conducted on Intel(R) Xeon(R) CPU E5–2696 v4 and 503 GB of memory. During the training phase, the average calculation time of a single training epoch is ∼ 13 h 28 min. For the testing phase, taking chromosome 16 as an example, the calculation time at 25-kb resolution, 10-kb resolution, and 1-kb resolution is 56 min 8 s, 4 h 18 min 44 s, and 52 h 34 min 41 s, respectively. Additional details on model training and hyperparameters are provided in File S1.

### Model evaluation

We started the evaluation process by making predictions on independent test sets using the best-trained model. The predicted correlation sub-matrices were then combined to form the intra-chromatin CM. The experimental chromatin interaction CMs at 25-kb resolution were considered as the ground truth. During evaluation, we used PCA provided by the sklearn package [50] to calculate the PC1 values of the two CMs. PC1 is generally considered to represent the A/B compartment information. Additionally, we discretized PC1 to obtain the chromatin compartment state, which was saved separately.

To assess model performance, we used several metrics, including MSE (Equation 6), MAE, SSIM (assessing the similarity of two CMs), and PSNR (measuring the quality score of the CMs) [51]. MAE, SSIM, and PSNR are defined by Equations  10–12:

(10) $$\begin{array}{c} MAE\left(Ŷ,Y\right)=\frac{1}{n}\sum_{i=1}^{n}\left|{\hat{y}}_{i}-y_{i}\right| \end{array}$$

 

(11) $$\begin{array}{c} SSIM\left(Ŷ,Y\right)=\frac{\left(2\mu_{Ŷ}\mu_{Y}+C_{1}\right)*\left(2\sigma_{ŶY}+C_{2}\right)}{\left(\mu_{Ŷ}^{2}+\mu_{Y}^{2}+C_{1}\right)*\left(\sigma_{Ŷ}^{2}+\sigma_{Y}^{2}+C_{2}\right)} \end{array}$$

 

(12) $$\begin{array}{c} PSNR\left(Ŷ,Y\right)=10*log_{10}\left(\frac{N}{MSE\left(Ŷ,Y\right)}\right) \end{array}$$

where Ŷ denotes the predicted CM, and Y represents the real CM. Furthermore, considering the chromatin compartmentalization information in the CMs, we evaluated their reproducibility using multiple PCC and GenomeDISCO score [52].

## Results

### Overview of COCOA

In this study, we proposed COCOA as a method for accurately predicting cell-type-specific chromatin compartment patterns at a fine-scale resolution by integrating epigenomic modification signals. COCOA only requires six epigenomic track signals as inputs, which are accessible for most tissues and cell lines in the ENCODE database [53]. The targets of COCOA are defined as the CMs of OE-normalized contact maps, allowing for the maximum retention of chromatin compartment pattern information. The COCOA framework connects these inputs and targets through binning, prediction, and combination operations (Figure 1A). Notably, in the binning process, we utilized the resolution-specific binning approach (*i.e.*, ${\mathrm{Bin}}_{\mathrm{epi}}={\mathrm{Bin}}_{\mathrm{corr}}$) instead of a single bin per genome site approach (*i.e.*, ${\mathrm{Bin}}_{\mathrm{epi}}={\mathrm{Bin}}_{\mathrm{corr}}*\mathrm{resolution}$). This choice greatly improves the practicality of COCOA.

We trained COCOA on Micro-C data of HFFc6 along with corresponding ChIP-seq data (Tables S1 and S2) using backpropagation algorithm. Specifically, COCOA first utilizes the bidirectional feature reconstruction module to calculate the 1D track features separately from two inputs. This step captures the intrinsic association present in the original epigenomic data in each direction (Figure 1B; see Method). Subsequently, the cross-attention fusion module integrates these 1D track feature maps with space contact features based on crossed attention mechanisms (see Method). Lastly, the residual feature reduction module decodes these contact features to generate predicted results, which are then combined into a complete CM (see Method). In addition, a composite loss function is employed to minimize the distance between the predicted targets and the ground truth.

### COCOA accurately predicts chromatin compartmentalization pattern

**Figure 2:**
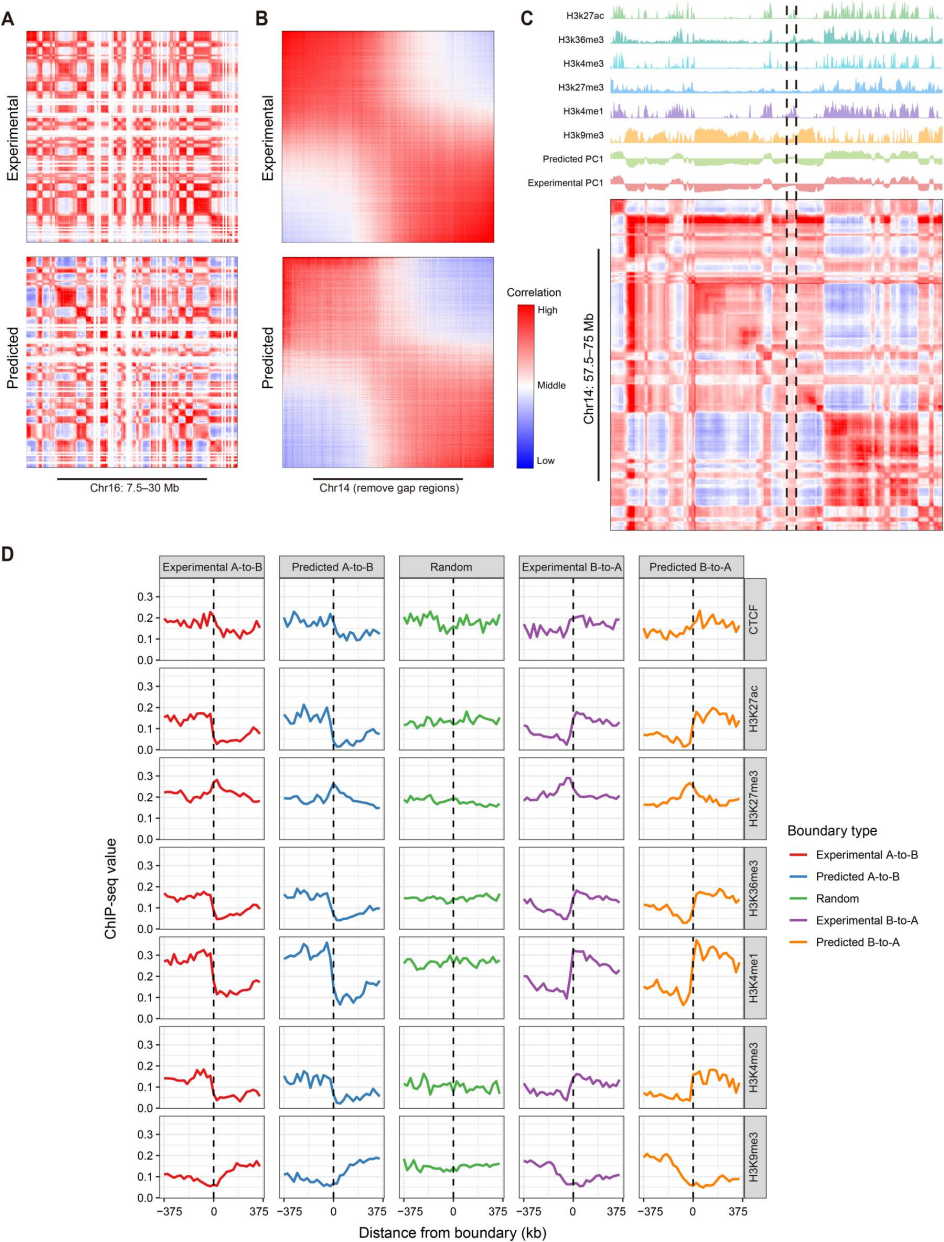
COCOA accurately predicts significant compartment patterns from epigenomic data **A**. A representative region illustrating the experimental and predicted CMs on test chromosomes. **B**. Heatmaps showing the experimental and predicted CMs, sorted according to their respective PC1 sizes. The predicted CM demonstrates consistency with the compartment patterns observed in the experimental CM. **C**. The predicted CM exhibits patterns that align precisely with the waveforms of histone modification signals. Within the region marked by the black dashed lines, COCOA is able to correct the pattern misclassified by the experimental data. **D**. Analysis of the shifts of epigenomic modification signals within 375-kb neighborhoods surrounding compartment boundaries in both experimental and predicted CMs. PC1, the first principal component; CTCF, CCCTC-binding factor.

To assess the performance of COCOA, we applied the trained COCOA model to randomly selected epigenomic data from the test sets (Chr12, Chr14, and Chr16) to generate predicted CMs. We considered the CM calculated from the Micro-C data and its PC1 as the experimental CM, which can be regarded as the ground truth for comparison. Heatmaps in **Figure 2**A and Figure S1A compare the typical genomic regions using the predicted and experimental CMs. The results demonstrate that the predicted CM generally exhibits the correct chromatin compartmentalization pattern. Furthermore, COCOA shows outstanding generative capacity in capturing subtle chromatin compartments. Notably, the predicted CM shows more pronounced interactions in dissimilar chromatin compartment blocks (blue blocks) compared with the experimental CM, while exhibiting partial over-reinforcement in identical chromatin compartment blocks (red blocks). We also computed the PC1 values of the predicted CM and the experimental CM using the sklearn package [50]. Subsequently, the CMs were sorted based on the size of their respective PC1 values (Figure 2B, Figure S1B). The results indicate that the modularity phenomenon of the predicted CM resembles the modularity patterns observed in the experimental CM. Similar results can be obtained by sorting the two CMs according to the PC1 size obtained from the predicted CM (Figure S2A). In addition, the predicted CM successfully captures the white band regions present in the experimental CM (Figure S1B).

To establish the biological significance of COCOA model predictions, we generated plots that illustrate the predicted CMs alongside the epigenomic signal tracks and the PC1 tracks. Figure S1C reveals that the predicted CM accurately shows plaid patterns of chromatin compartments, with each block of the plaid corresponding to a signal peak in the epigenomic data tracks. The PC1 values from the tracks of the experimental and predicted CMs also align precisely with these results. Importantly, COCOA can infer chromatin compartments that are consistent with the underlying epigenomic data but are not captured in the experimental CM (indicated by the black dotted lines in Figure 2C and Figure S2B). Moreover, we analyzed shifts of six epigenomic modification signals at compartment boundaries and randomly selected genomic loci, as done in previous studies [54]. Notably, we observed the consistent significant shifts of epigenomic modification signals within 375-kb neighborhoods around A/B compartment boundaries in both predicted and experimental CMs. These shifts were obviously different from randomly selected genomic loci (Figure 2D, Figure S1D). It is worth noting that shifts of partial epigenomic modification signals of the predicted CM generated by COCOA outperformed those of the experimental CM in capturing some compartment boundaries (*e.g.*, A2B boundary of H3K4me1 shown in Figure 2D).

### Genome-wide performance evaluation of COCOA

**Figure 3:**
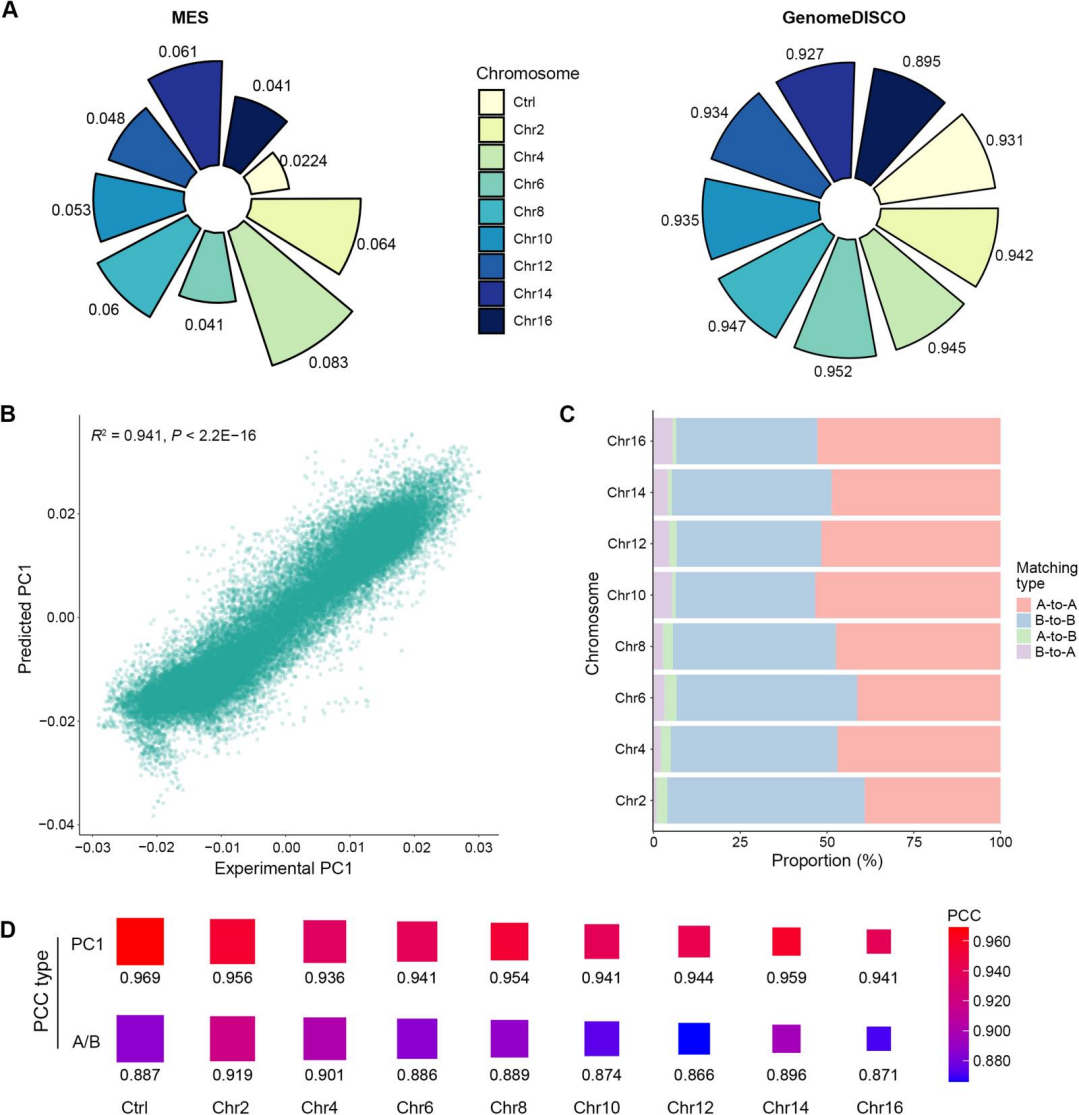
Genome-wide performance evaluation of COCOA in multiple metrics **A**. The MSE and genomeDISCO scores for COCOA on the test chromosomes. “Ctrl” represents the MSE and genomeDISCO scores between the two biological replicates. **B**. Scatter plot showing the high correlation between the PC1 values of the predicted and experimental CMs across the test sets. **C**. Proportion of compartment pattern matching between the predicted and experimental CMs. The red and blue bars represent the proportion of compartments that overlap between the predicted CM and experimental CM. The green and purple bars indicate the proportion of compartments that differ between the predicted and experimental CMs. **D**. Correlation analyses between the predicted and experimental CMs. The “Ctrl” column represents the PCCs between the two biological replicates. The “PC1” row represents the PCC of the PC1 values between the two CMs, and the “A/B” row represents the PCC of the binarized PC1 values between the two CMs. MSE, mean square error; PCC, Pearson correlation coefficient.

The performance of COCOA was quantitatively analyzed on genome-wide test sets. We calculated the MSE, MAE, PNSR, and SSIM scores to evaluate the robustness of error, signal-to-noise ratio, and structure similarity of COCOA on test sets. Compared to the score between two biological replicates, COCOA achieved competitive error and similarity scores on the test sets (**Figure 3**A, left panel; Table S3), exhibiting only minimal fluctuation with variations in the quality of the input data and the chromosome size. This stability indicates that COCOA performs consistently across different prediction scenarios (Figure 3A, left panel; Figure S3A; Table S3). In addition, we adopted GenomeDISCO scores, designed to assess the reproducibility of contact maps, to validate the biological significance of the predicted CM. As shown in Figure 3A (right panel) and Figure S3B, COCOA achieved high reproducibility between the predicted and experimental CMs.

As the CM contains abundant information regarding chromatin compartmentalization, we preformed correlation evaluations at the CM, PC1-value, and compartment-state levels. Figure 3B shows a scatter plot of the PC1 values between the predicted and experimental CMs across the test sets ($R^{2}=0.941,\;P<2.2E–16$). In Figure 3C, we observed that misclassification rates of A/B compartments were independent of chromosome length, and all remained below 10%. Furthermore, the PCCs of the PC1 values for paired predicted and experimental CMs were higher than 0.9, with the same trend observed for the PCCs of A/B compartment states (Figure 3D, Figure S3C). These PCCs are also consistent with the results of the correlation assessment between the two biological replicates. To evaluate COCOA’s performance on inferring deep chromatin compartmentalization information, we calculated the mean PCC for each column between the predicted CM and the ground truth. The results showed that the predicted CM achieved a high mean PCC when compared to the experimental CM (Figure S4).

### COCOA predicts chromatin compartmentalization changes to epigenomic perturbations

After confirming the accuracy of COCOA in inferring chromatin compartmentalization from epigenomic data, we used COCOA to perform *in silico* epigenomic perturbation experiments and assessed the impact of epigenomic signals on chromatin compartment pattern prediction.

**Figure 4:**
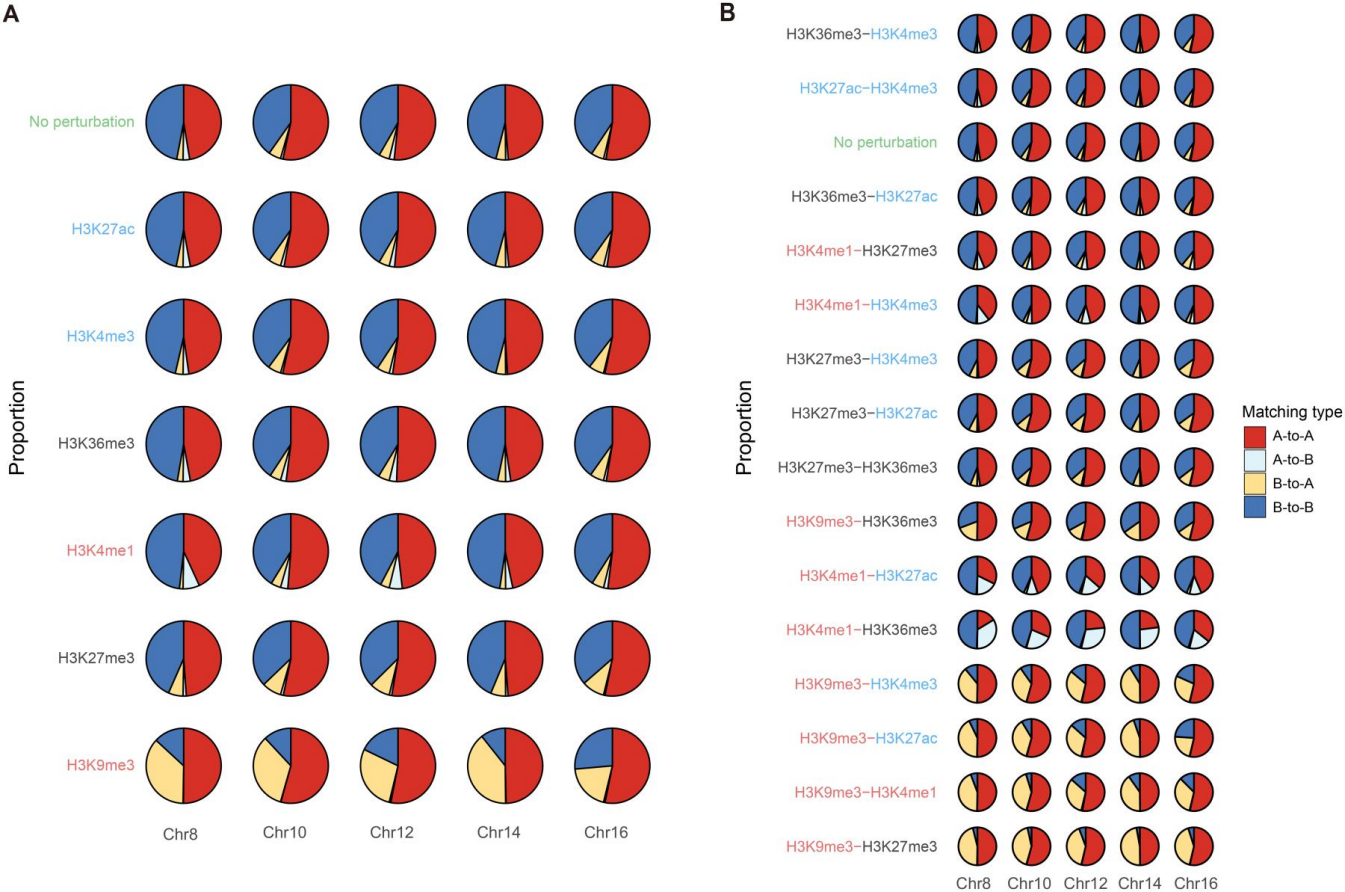
COCOA predicts compartment patterns for epigenomic perturbation experiments The row where green “No perturbation” (reference) indicates the comparison between the predicted CM and the experimental CM (ground truth) for the unperturbed data. Red, black, and blue labels in the vertical axis indicate high, medium, and low impact of the perturbed epigenomic signals on the chromatin compartment patterns, respectively. **A**. Proportion of compartment pattern matching between the predicted CM and the experimental CM from the one-perturbation epigenomic combinations. **B**. Proportion of compartment pattern matching between the predicted CM and the experimental CM from the two-perturbation epigenomic combinations.

In the single epigenomic signal perturbation (one-perturbation) experiments, we generated perturbed epigenomic data by setting one selected epigenomic signal to its minimum value while keeping other data unchanged. Subsequently, we predicted the corresponding CMs for the perturbed epigenomic data and compared them to their respective experimental CMs for the unperturbed data. The predicted results from one-perturbation experiments indicated that alerting H3K9me3 signal significantly influenced chromatin architecture, causing a substantial number of B-to-A compartment switches (**Figure 4**A). On the other hand, the perturbation of H3K4me1 signal led to a small proportion of A-to-B compartment switches. Perturbing other epigenomic signals (*i.e.*, H3K27ac, H3K27me3, H3K36me3, and H3K4me3) had no significant effect on chromatin compartment patterns (Figure 4A, Figure S5A).

To further analyze the contribution of individual epigenomic signals to the maintenance of chromatin compartment patterns, we conducted keep-one epigenomic signal perturbation (keep-one perturbation) experiments. In these experiments, we perturbed the epigenomic data by maintaining the selected epigenomic signal data while setting all other epigenomic signal data to their minimum values. Subsequently, we utilized COCOA to predict the chromatin compartment patterns for the perturbed data. The keep-one perturbation experiments revealed that the predicted CMs from H3K9me3 or H3K4me1 signal partially overlapped with their respective experimental CMs for the unperturbed data, while those predicted CMs from other epigenomic signals exhibited distinct differences from their respective experimental CMs (Figure S6A and B). This result reinforced the importance of H3K9me3 and H3K4me1 for predicting the status of chromatin architecture.

We next investigated the effects of two epigenomic signals on the chromatin compartmentalization through two epigenomic signal perturbation (two-perturbation) experiments. We observed that H3K9me3 and H3K4me1 signals play dominant roles in determining A/B compartment patterns. When H3K9me3 or H3K4me1 signal was perturbed along with H3K27ac, H3K27me3, H3K36me3, or H3K4me3 signal, the predicted chromatin compartment patterns exhibited significant changes compared to those for the unperturbed data (Figure S5B). Notably, perturbing the H3K9me3 signal gave rise to B-to-A compartment switches, while perturbing the H3K4me1 signal resulted in A-to-B compartment switches (Figure 4B). Simultaneous perturbations of H3K9me3 and H3K4me1 signals exhibited the greatest impact on the changes in chromatin compartment patterns among all the two-perturbation combinations.

Taken together, COCOA facilitates investigations into the role of epigenomic signals in determining chromatin compartmentalization prediction through *in silico* epigenomic perturbation experiments. Our results suggest that H3K9me3 and H3K4me1 signals are crucial for maintaining the chromatin compartment pattern prediction.

### COCOA shows robust performance of model predictions at different resolutions

**Figure 5:**
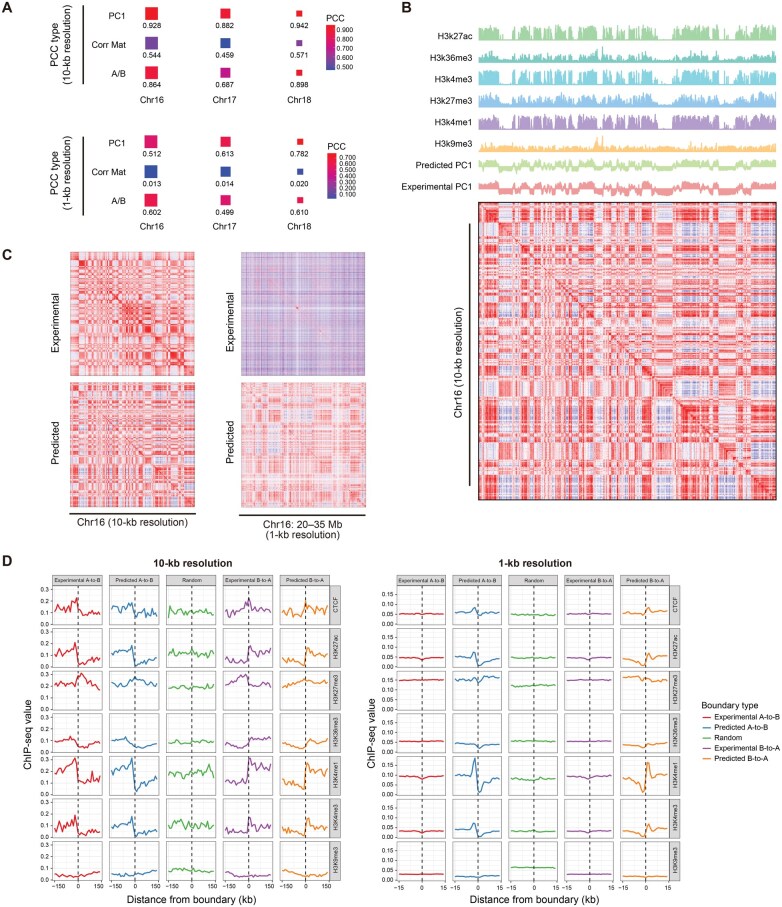
Prediction of resolution-specific compartment patterns by COCOA **A**. Correlation analyses between the predicted and experimental CMs at 10-kb and 1-kb resolutions, respectively. The “PC1” row represents the PCC of the PC1 values between the two CMs; the “Corr Mat” row represents the mean PCC for each column of the two CMs; and the “A/B” row represents the PCC of the binarized PC1 values between the two CMs. **B**. The predicted CM accurately captures the histone modification signal waveforms on Chr16 at 10-kb resolution. **C**. A typical region illustrating the predicted and experimental CMs at 10-kb and 1-kb resolutions, respectively. At 1-kb resolution, the experimental CM exhibits high noise levels and lacks recognizable plaid patterns, while the predicted CM demonstrates clear plaid patterns. **D**. Analysis of the shifts of histone modification signals within 150-kb and 15-kb neighborhoods surrounding compartment boundaries in both predicted and experimental CMs at 10-kb and 1-kb resolutions, respectively. At 10-kb resolution, both predicted and experimental CMs display meaningful shifts, whereas at 1-kb resolution, the experimental CM approaches random results, while the predicted CM still shows significant biological shifts.

To evaluate the performance of COCOA at different resolutions, we used the model trained at 25-kb resolution to predict the fine-scale CM using resolution-specific inputs. We first used the trained model to predict 10-kb-resolution CMs for Chr16, Chr17, and Chr18 datasets and evaluated the performance of the predictions. The results showed that COCOA achieved consistent and competitive scores on all three test sets (Table S4). We further evaluated the correlations between the predicted compartment patterns and the ground-truth patterns. The predicted 10-kb-resolution CM was highly correlated with the experimental CM (**Figure 5**A). A similar high correlation of A/B compartment states was observed between the predicted and experimental CMs. The compartment misclassification rates were all below 0.2, indicating that COCOA generates CMs containing reliable chromatin compartment information (Figure S7A). The predicted CM exhibited similar plaid patterns to the experimental CM, corresponding well with the epigenomic signals (Figure 5B and C). In addition, the modularity of the predicted CM aligned with that of the experimental CM on the whole (Figure S7C). We also observed consistent and significant shifts of epigenomic modification signals within 150-kb neighborhoods around A/B compartment boundaries in both predicted and experimental CMs, which are distinguishable from randomly selected genomic loci (Figure 5D).

To evaluate COCOA’s performance at ultra-high resolution, we employed the model trained at 25-kb resolution to predict the 1-kb-resolution CMs for Chr16, Chr17, and Chr18 datasets. Similar to the evaluation at 10-kb resolution, we assessed the performance metrics and correlations. The results showed that COCOA achieved robust performance across a wide range of scores, but obtained scores close to 0 for PCCs at the CM level (Figure 5A; Table S4). This may be attributed to the sparsity of the deeply-sequenced experimental CM at ultra-high resolution (*i.e.*, ∼ 2.6–4.5 billion uniquely mapped reads with ∼ 150× coverage per nucleosome) [47], which is challenging to define as the ground truth. As the CM size increases, the mean error evaluation narrows the gap, producing similar scores. However, using PCA-based correlations or compartment misclassification rates, we can partially mitigate the sparsity issue and obtain reliable scores (Figure S7B). Therefore, we visualized the experimental and predicted CMs by heatmaps (Figure 5C). We found that the experimental CM showed vaguely visible plaid patterns and was filled with noise-induced thin lines. In contrast, the predicted CM remained consistent with these fuzzy patterns but displayed more apparent compartmentalization patterns (Figure 5C, Figure S7D). Moreover, we investigated the shifts of epigenomic modification signals within 15-kb neighborhoods around A/B compartment boundaries in both the predicted and experimental CMs. Surprisingly, the shifts observed in the experimental CM were similar to those in randomly selected genome loci, while the predicted CM showed significant and biologically meaningful shifts (Figure 5D).

### COCOA accurately predicts cell-type-specific chromatin compartment patterns

**Figure 6:**
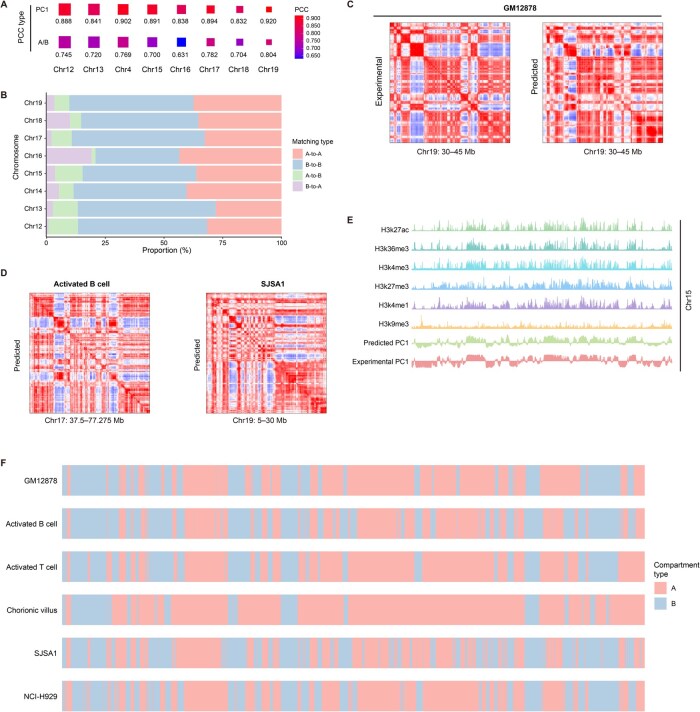
Prediction of cell-type-specific compartment patterns by COCOA **A**. Correlation analyses between the predicted and experimental CMs on the GM12878 dataset. The “PC1” row represents the PCC of the PC1 values between the two CMs, and the “A/B” row represents the PCC of the binarized PC1 values between the two CMs. **B**. Proportion of compartment pattern matching between the predicted and experimental CMs on the GM12878 dataset. **C**. A typical region illustrating the predicted and experimental CMs on the GM12878 dataset. **D**. Example regions illustrating the predicted CMs on the datasets of activated B cells and SJSA1 cells. **E**. Precisely matching of the predicted CMs with the waveforms of histone modification signals on Chr15 in the GM12878 dataset. **F**. Systematic comparison of chromatin compartment statuses on Chr15 across the datasets of GM12878, activated B cells, activated T cells, chorionic villus cells, SJSA1 cells, and NCI-H929 cells.

Because epigenomic data are cell-type-specific, we tested whether COCOA can accurately predict chromatin compartment patterns in different cell types. We first applied COCOA to the GM12878 dataset and generated the predicted CMs for multiple chromosomes. The corresponding experimental CMs obtained from the Hi-C data of GM12878 [6] served as the ground truth for comparison. The results indicated that high correlations were observed between the predicted and experimental CMs and between the PC1 values of the two CMs (**Figure 6**A, Figure S8A). Figure 6B showed that the compartment misclassification rates were all below 20%. Furthermore, the predicted CM presented the plaid patterns consistent with those of the experimental CM, achieving stable and competitive scores in terms of both error and the image similarity (Figure 6C; Table S5). Taken together, these results suggest that COCOA reliably predicts the cell-type-specific chromatin compartment patterns.

Having established COCOA’s capability to predict CMs across diverse cell types, we proceeded to predict CMs in five additional cell types (*i.e.*, SJSA1 cells, NCI-H929 cells, activated B cells, activated T cells, and chorionic villus cells) representing tissues, diseases, and primary cells where chromatin conformation had not been sequenced. The predicted CMs for the five datasets displayed obvious plaid patterns (Figure 6D, Figure S8B). The predicted A/B compartments in SJSA1, NCI-H929, and activated B cells were clearly defined and noise-free, while the predicted CMs of activated T cells and chorionic villus cells displayed slightly diminished performance. To gain insight into the effects of chromatin compartmentalization in disease and differentiation, we systematically compared the patterns across different cell types. Using the predicted CM from GM12878 data as a benchmark, we examined commonalities and differences in chromatin region patterns based on histone modification track information (Figure 6E). Similar chromatin compartment patterns were observed between GM12878 and most other cells, albeit with variations in certain regions (Figure 6F). Notably, activated B cells, being immune cells akin to GM12878, exhibited a comparable compartment pattern. Similarly, activated T cells demonstrated an analogous pattern. In contrast, SJSA1, NCI-H929, and chorionic villus cells exhibited distinct compartment patterns.

## Discussion

In this study, we developed a deep neural network framework, COCOA, which incorporates six types of epigenomic modification signals to accurately predict fine-scale-resolution chromatin compartment patterns. These epigenomic signal data are readily accessible in ENCODE database [53] for various cell lines, in vitro differentiated cells, primary cells, and tissues. To process the raw epigenomic data, we employed resolution-specific preprocessing to bin the data into mated inputs from different genomic positions. COCOA then uses the bidirectional feature reconstruction module to extract track features from these mated inputs, then fuses these track features to contact features using the cross-attention fusion module. Eventually, these contact features are converted to chromatin compartment patterns by the residual feature reduction module. COCOA predicts directly long-range chromatin compartment patterns without considering short-range interactions [28,29,35]. Our results demonstrate that COCOA accurately predicts the same chromatin compartment patterns as the experimental CM, with consistent epigenomic signal shifts of these patterns (Figure 2D). During model evaluation, COCOA achieves excellent performance with robust reproducibility scores on the test sets. Furthermore, the predicted CM and its PC1 values show a high correlation with the experimental CM and its PC1 values. The compartment misclassification rates of the predicted CM remain below 10% and are independent of chromosome length.

With COCOA’s accurate prediction of chromatin compartmentalization, it becomes possible to perform *in silico* epigenomic perturbation to study the influence of histone modification signals on chromatin compartmentalization prediction. By generating predicted CMs using different perturbed epigenomic data, we found that H3K9me3 has strong impact on chromatin compartment pattern prediction, followed by H3K4me1. In contrast, H3K27me3 and H3K36me3 have a moderate level of impact, and H3K27ac and H3K4me3 have low impact. Interestingly, COCOA predicted that perturbation of H3K9me3 signal led to compartment B-to-A changes, while perturbation of H3K4me1 signal resulted in A-to-B compartment switches. Additionally, H3K9me3 and H3K4me1signals play dominant roles in determining chromatin compartment patterns when they are perturbed together with other epigenomic signals in two-perturbation experiments. These findings contrast with a previous study by Zheng et al. [55], which identifies H3K27ac and H3K36me3 as the top two predictive histone marks for CoRNN. This inconsistency arises from substantial differences in the target (global contact pattern for COCOA *versus* A/B compartment for CoRNN), as well as variations in question formulation, such as regression (COCOA) *versus* classification (CoRNN).

Furthermore, we explored the performance of COCOA’s predictions across different resolutions and cell types. For prediction at 10-kb resolution, COCOA exhibited the same outstanding performance as predicted at the trained 25-kb resolution. Recognizing the significance of high resolution in chromatin interaction data analysis, we investigated whether COCOA can make good prediction at 1-kb resolution. Unfortunately, even with a high sequencing depth [47], the experimental CM at 1-kb resolution contains excessive noise lines and barely discernible plaid patterns. Therefore, we analyzed histone modification shifts at compartment boundaries and mapped heatmaps of the predicted CM at different genome ranges. Surprisingly, the predicted CM displayed clearer plaid patterns and exhibited more biologically meaningful shifts compared to the experimental CM and randomly selected loci. We then evaluated the performance of COCOA in predicting cell-type-specific compartment patterns. Using validated Hi-C data of GM12878, our results demonstrated that COCOA can correctly infer chromatin compartment patterns from epigenomic data on unseen cell lines.

While this work presents promising results, it also has several potential areas for improvements. Firstly, as a data-driven approach, COCOA relies on moderately good-quality training sets to achieve high performance by incorporating potential information from bidirectional epigenomic data. In addition, we observed that the transfer capacity of COCOA in cross-cell-line experiments is affected by the epigenomic data quality. Developing new data processing schemes may prove beneficial in solving this issue. Secondly, in challenging task such as high-volume fine-scale-resolution CM prediction and *in silico* epigenomic signal perturbation experiments, COCOA requires significant run time and substantial computational resources. To alleviate this computational burden, parallel CM generation and distributed implementations can be explored as feasible approaches [56]. Thirdly, we also preliminarily explored the influences of histone modification signals on A/B chromatin compartmentalization in HFF datasets by *in silico* epigenomic perturbation experiments. However, more systematically studying the combined impacts of epigenomic modifications in relation to complex chromatin compartmentalization on different cell lines would benefit from further experimental evidence. Lastly, COCOA’s predictions for fine-scale chromatin compartmentalization information in diseases, tissues, and primary cells have not been thoroughly explored. In the future, it would be interesting to explore the impact of the chromatin compartment alteration on cell differentiation and disease occurrence by integrating epigenomics data with other omics and phenotypic data.

## Code availability

The source code has been implemented in Python and can be freely accessed on GitHub (https://github.com/onlybugs/COCOA). The source code has also been submitted to BioCode at the National Genomics Data Center, Beijing Institute of Genomics, Chinese Academy of Sciences / China National Center for Bioinformation (BioCode: BT007498), which is publicly accessible at https://ngdc.cncb.ac.cn/biocode/tools/BT007498.

## Supplementary Material

qzae091_Supplementary_Data

## CRediT author statement

**Kai Li:** Conceptualization, Methodology, Software, Data curation, Visualization, Formal analysis, Writing – original draft, Writing – review & editing. **Ping Zhang:** Conceptualization, Writing – review & editing. **Jinsheng Xu:** Data curation. **Zi Wen:** Data curation. **Junying Zhang:** Data curation. **Zhike Zi:** Writing – review & editing, Resources, Conceptualization. **Li Li:** Writing – review & editing, Project administration, Resources. All authors have read and approved the final manuscript.

## References

[1] Ke Y , Xu Y, Chen X, Feng S, Liu Z, Sun Y, et al 3D chromatin structures of mature gametes and structural reprogramming during mammalian embryogenesis. Cell 2017;170:367–81.e20. 28709003 10.1016/j.cell.2017.06.029

[2] Li T , Li R, Dong X, Shi L, Lin M, Peng T, et al Integrative analysis of genome, 3D genome, and transcriptome alterations of clinical lung cancer samples. Genomics Proteomics Bioinformatics 2021;19:741–53. 34116262 10.1016/j.gpb.2020.05.007 PMC9170781

[3] Lieberman-Aiden E , van Berkum NL, Williams L, Imakaev M, Ragoczy T, Telling A, et al Comprehensive mapping of long-range interactions reveals folding principles of the human genome. Science 2009;326:289–93. 19815776 10.1126/science.1181369 PMC2858594

[4] Hsieh THS , Weiner A, Lajoie B, Dekker J, Friedman N, Rando OJ. Mapping nucleosome resolution chromosome folding in yeast by Micro-C. Cell 2015;162:108–19. 26119342 10.1016/j.cell.2015.05.048 PMC4509605

[5] Deshpande AS , Ulahannan N, Pendleton M, Dai X, Ly L, Behr JM, et al Identifying synergistic high-order 3D chromatin conformations from genome-scale nanopore concatemer sequencing. Nat Biotechnol 2022;40:1488–99. 35637420 10.1038/s41587-022-01289-z

[6] Rao SS , Huntley MH, Durand NC, Stamenova EK, Bochkov ID, Robinson JT, et al A 3D map of the human genome at kilobase resolution reveals principles of chromatin looping. Cell 2014;159:1665–80. 25497547 10.1016/j.cell.2014.11.021 PMC5635824

[7] Nora EP , Lajoie BR, Schulz EG, Giorgetti L, Okamoto I, Servant N, et al Spatial partitioning of the regulatory landscape of the X-inactivation centre. Nature 2012;485:381–5. 22495304 10.1038/nature11049 PMC3555144

[8] Dixon JR , Selvaraj S, Yue F, Kim A, Li Y, Shen Y, et al Topological domains in mammalian genomes identified by analysis of chromatin interactions. Nature 2012;485:376–80. 22495300 10.1038/nature11082 PMC3356448

[9] Vian L , Pękowska A, Rao SSP, Kieffer-Kwon KR, Jung S, Baranello L, et al The energetics and physiological impact of cohesin extrusion. Cell 2018;175:292–4. 30241609 10.1016/j.cell.2018.09.002 PMC6251724

[10] Goel VY , Huseyin MK, Hansen AS. Region capture Micro-C reveals coalescence of enhancers and promoters into nested microcompartments. Nat Genet 2023;55:1048–56. 37157000 10.1038/s41588-023-01391-1 PMC10424778

[11] Simonis M , Klous P, Splinter E, Moshkin Y, Willemsen R, de Wit E, et al Nuclear organization of active and inactive chromatin domains uncovered by chromosome conformation capture-on-chip (4C). Nat Genet 2006;38:1348–54. 17033623 10.1038/ng1896

[12] Spracklin G , Abdennur N, Imakaev M, Chowdhury N, Pradhan S, Mirny LA, et al Diverse silent chromatin states modulate genome compartmentalization and loop extrusion barriers. Nat Struct Mol Biol 2023;30:38–51. 36550219 10.1038/s41594-022-00892-7 PMC9851908

[13] Haws SA , Simandi Z, Barnett RJ, Phillips-Cremins JE. 3D genome, on repeat: higher-order folding principles of the heterochromatinized repetitive genome. Cell 2022;185:2690–707. 35868274 10.1016/j.cell.2022.06.052 PMC10225251

[14] Feng Y , Wang Y, Wang X, He X, Yang C, Naseri A, et al Simultaneous epigenetic perturbation and genome imaging reveal distinct roles of H3K9me3 in chromatin architecture and transcription. Genome Biol 2020;21:296. 33292531 10.1186/s13059-020-02201-1 PMC7722448

[15] Nichols MH , Corces VG. Principles of 3D compartmentalization of the human genome. Cell Rep 2021;35:109330. 34192544 10.1016/j.celrep.2021.109330 PMC8265014

[16] Wen Z , Zhang W, Zhong Q, Xu J, Hou C, Qin ZS, et al Extensive chromatin structure–function associations revealed by accurate 3D compartmentalization characterization. Front Cell Dev Biol 2022;10:845118. 35517497 10.3389/fcell.2022.845118 PMC9062080

[17] Harris HL , Gu H, Olshansky M, Wang A, Farabella I, Eliaz Y, et al Chromatin alternates between A and B compartments at kilobase scale for subgenic organization. Nat Commun 2023;14:3303. 37280210 10.1038/s41467-023-38429-1 PMC10244318

[18] Rao SSP , Huang SC, Glenn St Hilaire B, Engreitz JM, Perez EM, Kieffer-Kwon KR, et al Cohesin loss eliminates all loop domains. Cell 2017;171:305–20.e24. 28985562 10.1016/j.cell.2017.09.026 PMC5846482

[19] Fortin JP , Hansen KD. Reconstructing A/B compartments as revealed by Hi-C using long-range correlations in epigenetic data. Genome Biol 2015;16:180. 26316348 10.1186/s13059-015-0741-y PMC4574526

[20] Schmitt AD , Hu M, Ren B. Genome-wide mapping and analysis of chromosome architecture. Nat Rev Mol Cell Biol 2016;17:743–55. 27580841 10.1038/nrm.2016.104 PMC5763923

[21] LeCun Y , Bengio Y, Hinton G. Deep learning. Nature 2015;521:436–44. 26017442 10.1038/nature14539

[22] Yang JY , Chang JM. Pattern recognition of topologically associating domains using deep learning. BMC Bioinformatics 2022;22:634. 36482308 10.1186/s12859-022-05075-1 PMC9732975

[23] Soler-Vila P , Cuscó P, Farabella I, Di Stefano M, Marti-Renom MA. Hierarchical chromatin organization detected by TADpole. Nucleic Acids Res 2020;48:e39. 32083658 10.1093/nar/gkaa087 PMC7144900

[24] Zhang S , Plummer D, Lu L, Cui J, Xu W, Wang M, et al *DeepLoop* robustly maps chromatin interactions from sparse allele-resolved or single-cell Hi-C data at kilobase resolution. Nat Genet 2022;54:1013–25. 35817982 10.1038/s41588-022-01116-w PMC10082397

[25] Zhang Y , Blanchette M. Reference panel guided topological structure annotation of Hi-C data. Nat Commun 2022;13:7426. 36460680 10.1038/s41467-022-35231-3 PMC9718747

[26] Wang B , Liu K, Li Y, Wang J. DFHiC: a dilated full convolution model to enhance the resolution of Hi-C data. Bioinformatics 2023;39:btad211. 37084258 10.1093/bioinformatics/btad211 PMC10166584

[27] Zhang Y , An L, Xu J, Zhang B, Zheng WJ, Hu M, et al Enhancing Hi-C data resolution with deep convolutional neural network HiCPlus. Nat Commun 2018;9:750. 29467363 10.1038/s41467-018-03113-2 PMC5821732

[28] Schwessinger R , Gosden M, Downes D, Brown RC, Oudelaar AM, Telenius J, et al DeepC: predicting 3D genome folding using megabase-scale transfer learning. Nat Methods 2020;17:1118–24. 33046896 10.1038/s41592-020-0960-3 PMC7610627

[29] Fudenberg G , Kelley DR, Pollard KS. Predicting 3D genome folding from DNA sequence with Akita. Nat Methods 2020;17:1111–7. 33046897 10.1038/s41592-020-0958-x PMC8211359

[30] Yang R , Das A, Gao VR, Karbalayghareh A, Noble WS, Bilmes JA, et al Epiphany: predicting Hi-C contact maps from 1D epigenomic signals. Genome Biol 2023;24:134. 37280678 10.1186/s13059-023-02934-9 PMC10242996

[31] Zhang R , Zhou T, Ma J. Multiscale and integrative single-cell Hi-C analysis with Higashi. Nat Biotechnol 2022;40:254–61. 34635838 10.1038/s41587-021-01034-y PMC8843812

[32] Xiong K , Zhang R, Ma J. scGHOST: identifying single-cell 3D genome subcompartments. Nat Methods 2024;21:814–22. 38589516 10.1038/s41592-024-02230-9 PMC11127718

[33] Zhang S , Chasman D, Knaack S, Roy S. *In silico* prediction of high-resolution Hi-C interaction matrices. Nat Commun 2019;10:5449. 31811132 10.1038/s41467-019-13423-8 PMC6898380

[34] Zhou J. Sequence-based modeling of three-dimensional genome architecture from kilobase to chromosome scale. Nat Genet 2022;54:725–34. 35551308 10.1038/s41588-022-01065-4 PMC9186125

[35] Tan J , Shenker-Tauris N, Rodriguez-Hernaez J, Wang E, Sakellaropoulos T, Boccalatte F, et al Cell-type-specific prediction of 3D chromatin organization enables high-throughput *in silico* genetic screening. Nat Biotechnol 2023;41:1140–50. 36624151 10.1038/s41587-022-01612-8 PMC10329734

[36] Reiff SB , Schroeder AJ, Kırlı K, Cosolo A, Bakker C, Mercado L, et al The 4D nucleome data portal as a resource for searching and visualizing curated nucleomics data. Nat Commun 2022;13:2365. 35501320 10.1038/s41467-022-29697-4 PMC9061818

[37] Abdennur N , Mirny LA. Cooler: scalable storage for Hi-C data and other genomically labelled arrays. Bioinformatics 2020;36:311–6. 31290943 10.1093/bioinformatics/btz540 PMC8205516

[38] Kim TH , Dekker J. ChIP-seq. Cold Spring Harb Protoc 2018;2018:363–8. 10.1101/pdb.prot082644 29717046

[39] Colwell J. Expanding the scope of ENCODE. Cancer Discov 2016;6:OF4. 10.1158/2159-8290.CD-NB2016-020 26880692

[40] Li K , Zhang P, Wang Z, Shen W, Sun W, Xu J, et al iEnhance: a multi-scale spatial projection encoding network for enhancing chromatin interaction data resolution. Brief Bioinform 2023;24:bbad245. 37381618 10.1093/bib/bbad245

[41] Wang X , Qin Y, Wang Y, Xiang S, Chen H. ReLTanh: an activation function with vanishing gradient resistance for SAE-based DNNs and its application to rotating machinery fault diagnosis. Neurocomputing 2019;363:88–98.

[42] Dai Y , Gieseke F, Oehmcke S, Wu Y, Barnard K. Attentional feature fusion. Proceedings of the IEEE/CVF Winter Conference on Applications of Computer Vision 2021:3560–9.

[43] He K , Zhang X, Ren S, Sun J. Deep residual learning for image recognition. 2016 IEEE Conference on Computer Vision and Pattern Recognition 2016:770–8.

[44] Ioffe S , Szegedy C. Batch Normalization: accelerating deep network training by reducing internal covariate shift. Proceedings of the 32nd International Conference on International Conference on Machine Learning 2015;37:448–56.

[45] Johnson J , Alahi A, Li FF. Perceptual losses for real-time style transfer and super-resolution. In: Leibe B, Matas J, Sebe N, Welling M, editors. Computer Vision – ECCV 2016. Cham: Springer; 2016, p.694–711.

[46] Gatys LA , Ecker AS, Bethge M. A neural algorithm of artistic style. arXiv 2015;1508.06576.

[47] Krietenstein N , Abraham S, Venev SV, Abdennur N, Gibcus J, Hsieh TS, et al Ultrastructural details of mammalian chromosome architecture. Mol Cell 2020;78:554–65.e7. 32213324 10.1016/j.molcel.2020.03.003 PMC7222625

[48] Paszke A , Gross S, Massa F, Lerer A, Bradbury J, Chanan G, et al PyTorch: an imperative style, high-performance deep learning library. Advances in Neural Information Processing Systems 2019.

[49] Kingma DP , Ba J. Adam: a method for stochastic optimization. 3rd International Conference for Learning Representations 2014.

[50] Pedregosa F , Varoquaux G, Gramfort A, Michel V, Thirion B, Grisel O, et al Scikit-learn: machine learning in Python. J Mach Learn Res 2011;12:2825–30.

[51] Wang Z , Bovik AC, Sheikh HR, Simoncelli EP. Image quality assessment: from error visibility to structural similarity. IEEE Trans Image Process 2004;13:600–12. 15376593 10.1109/tip.2003.819861

[52] Yang T , He X, An L, Li Q. Methods to assess the reproducibility and similarity of Hi-C data. Methods Mol Biol 2022;2301:17–37. 34415529 10.1007/978-1-0716-1390-0_2

[53] Luo Y , Hitz BC, Gabdank I, Hilton JA, Kagda MS, Lam B, et al New developments on the encyclopedia of DNA elements (ENCODE) data portal. Nucleic Acids Res 2020;48:D882–9. 31713622 10.1093/nar/gkz1062 PMC7061942

[54] Xiong K , Ma J. Revealing Hi-C subcompartments by imputing inter-chromosomal chromatin interactions. Nat Commun 2019;10:5069. 31699985 10.1038/s41467-019-12954-4 PMC6838123

[55] Zheng S , Thakkar N, Harris HL, Liu S, Zhang M, Gerstein M, et al. Predicting A/B compartments from histone modifications using deep learning. iScience 2024;27:109570. 38646172 10.1016/j.isci.2024.109570 PMC11031843

[56] Hu Y , Ma W. EnHiC: learning fine-resolution Hi-C contact maps using a generative adversarial framework. Bioinformatics 2021;37:i272–9. 34252966 10.1093/bioinformatics/btab272 PMC8382278

