# Supplementary material for: COCOA: A Framework for Fine-scale Mapping of Cell-type-specific Chromatin Compartments Using Epigenomic Information
Source: Genomics Proteomics Bioinformatics. 2024 Dec 26;22(6):qzae091. doi: 10.1093/gpbjnl/qzae091 (PMC11993304; doi:10.1093/gpbjnl/qzae091)
Supplement: qzae091_Supplementary_Data [file qzae091_supplementary_data.zip › qzae091_Supplementary_Data/File S1.docx]

**File S1 Supplementary notes**

**Model training and hyper-parameters details**

All the training and validation processes were conducted on Intel(R) Xeon(R) CPU E5–2696 v4 and 503 GB of memory. The model is trained with a batch size of 16 for 120 epochs and adopts the Adam optimizer with an initial learning rate of 5E–4 (lr_init=5E–4). In addition, we employed early stopping to prevent overfitting and used the step learning rate scheduler [lr_n= lr_init*dr^(n/10), where n denotes the current epoch, dr is decay ratio].

**Model training and prediction times details**

During the training phase, the CPU is in a high load state outside the I/O period, and the peak memory reaches ~ 80 G. The average calculation time of a single training epoch is ~ 13 h 28 min, and the average calculation time of each minibatch is 17.57 s. For the testing phase, the total inferring time of chromosome 16 at 25 kb resolution, 10 kb resolution, and 1 kb resolution is 56 m 8 s, 4 h 18 m 44 s, and 52 h 34 m 41 s, respectively.
