# Supplementary material for: COCOA: A Framework for Fine-scale Mapping of Cell-type-specific Chromatin Compartments Using Epigenomic Information
Source: Genomics Proteomics Bioinformatics. 2024 Dec 26;22(6):qzae091. doi: 10.1093/gpbjnl/qzae091 (PMC11993304; doi:10.1093/gpbjnl/qzae091)
Supplement: qzae091_Supplementary_Data [file qzae091_supplementary_data.zip › qzae091_Supplementary_Data/supplementary material captions.docx]

**Supplementary material**

**File S1 Supplementary notes**

**Figure S1 COCOA precisely infers biological compartment patterns from epigenomic data**

**A.** Representative region illustrating predicted and experimental CM on Chr14: 30-50Mb. **B.** Heatmaps of the experimental CM and the predicted CM, sorted according to their respective PC1 sizes. **C.** The predicted CM exhibits patters that align precisely with the waveform of histone modification signal. **D.** Representative shifts in modification signals within 375 kb neighbourhoods surrounding compartment boundaries in both predicted and experimental CMs.

**Figure S2 COCOA enables accurate prediction of compartment pattern**

**A.** Heatmaps of the experimental CM and the predicted CM on Chr 14 and Chr 16, sorted according to the same PC1 sizes. **B.** The predicted CM exhibits patters that align precisely with the waveform of histone modification signal. Within the region marked by the black dashed line, COCOA is able to correct the pattern misclassified by the experiment.

**Figure S3 COCOA enables reliable cell-type-specific prediction of compartment pattern**

**A.** and **B.** Scatterplot showing the MSE and genomeDISCO scores for COCOA on the test chromosomes. **C.** Correlation coefficient scatterplot between the predicted CM and experimental CM on the test chromosomes. The “PC1” row represents the correlation of the PC1 of the CM and the “A/B” row represents the correlation after binarization of PC1.

**Figure S4 Correlation coefficient between the predicted** $\mathbf{CM}$ **and the experimental** $\mathbf{CM}$

The “Ctrl” column represents the correlation scores between the two biological replicates. The “Corr Mat” row represents the average correlation coefficient of each column of the CM.

**Figure S5 COCOA performs the results of the epigenomic one-perturbation and the two-perturbation experiments**

The row where green “No perturbation” (Reference) indicates the comparison between the predicted CM and ground truth for unperturbed data. Red, black, and blue fonts in the vertical axis labels indicate high, medium, and low impact of the perturbed epigenomic signal on the chromatin compartment patterns, respectively. **A.** The average correlation coefficients between the predicted CM from one-perturbation experiments and the experimental CM from the unperturbed data. The corresponding PC1 correlation coefficients are also shown for different test chromosomes. **B.** The similar PC1 correlation coefficients from two-perturbation experiments.

**Figure S6 COCOA performs the results of the keep-one perturbation experiments**

**A.** The average correlation coefficients between the predicted CM from keep-one perturbation experiments and the experimental CM from the unperturbed data. The corresponding PC1 correlation coefficients are also shown for different test chromosomes. **B.** Proportion of the compartment pattern matching between the predicted CM and the ground-truth CM from the keep-one perturbation experiments.

**Figure S7 COCOA achieves reliable performance across different resolutions**

**A.** Proportion of compartment pattern matching between the predicted CM and the experimental CM at 10 kb resolution. The red and blue bars represent the proportion of compartments that overlap between the predicted CM and experimental CM. The green and purple bars indicate the proportion of compartments that differ between the predicted CM and experimental CM. **B.** Proportion of compartment pattern matching between the predicted CM and the experimental CM at 1 kb resolution. **C.** Heatmaps of the experimental CM and the predicted CM, sorted according to their respective PC1 (Left panel) and the same PC1 (Right panel) sizes on Chr 16 at 10 kb resolution. **D.** Typical region of the predicted CM and the experimental CM at 1 kb resolutions.

**Figure S8 COCOA enables reliable cell-type-specific prediction of compartment pattern**

**A.** Correlation coefficient between the predicted CM and experimental CM on GM12878 datasets. The “Corr Mat” row represents the average correlation coefficient of each column of the CM. **B.** Example regions illustrating the predicted CM patterns on chorionic villus, activated T cell and NCI-H929 datasets

**Table S1 Micro-C and Hi-C data**

**Table S2 ChIP-seq data**

**Table S3 Summary table (performance evaluation)**

**Table S4 Summary table (multiple resolution)**

**Table S5 Summary table (cell-type-specific prediction)**
