# Supplementary material for: COCOA: A Framework for Fine-scale Mapping of Cell-type-specific Chromatin Compartments Using Epigenomic Information
Source: Genomics Proteomics Bioinformatics. 2024 Dec 26;22(6):qzae091. doi: 10.1093/gpbjnl/qzae091 (PMC11993304; doi:10.1093/gpbjnl/qzae091)
Supplement: qzae091_Supplementary_Data [file qzae091_supplementary_data.zip › qzae091_Supplementary_Data/Table S1.docx]

**Table S1 Micro-C and Hi-C data**

| **Type** | **Data** | **Accession number** |
| --- | --- | --- |
| Micro-C | HFFc6 | 4DNESWST3UBH |
| Hi-C | GM12878 | 4DNFIXP4QG5B |
