# Supplementary material for: COCOA: A Framework for Fine-scale Mapping of Cell-type-specific Chromatin Compartments Using Epigenomic Information
Source: Genomics Proteomics Bioinformatics. 2024 Dec 26;22(6):qzae091. doi: 10.1093/gpbjnl/qzae091 (PMC11993304; doi:10.1093/gpbjnl/qzae091)
Supplement: qzae091_Supplementary_Data [file qzae091_supplementary_data.zip › qzae091_Supplementary_Data/Table S2.docx]

**Table S2 ChIP-seq data**

| **Type** | **HFFc6** | **GM12878** | **Activated T cell** | **Activated B cell** | **NCI-H929** | **SJSA1** | **Chorionic villus** |
| --- | --- | --- | --- | --- | --- | --- | --- |
| H3K4me3 | ENCFF598MLG | ENCFF012DMX | ENCFF641ONK | ENCFF399LTS | ENCFF754JLX | ENCFF596HJB | ENCFF548VTO |
| H3K27ac | ENCFF644GJB | ENCFF798KYP | ENCFF169DFQ | ENCFF490TAG | ENCFF612UYR | ENCFF005KFY | ENCFF884SVO |
| H3K27me3 | ENCFF854VTY | ENCFF677PYB | ENCFF328DSH | ENCFF394DBD | ENCFF588PCB | ENCFF548QPW | ENCFF840RKG |
| H3K4me1 | ENCFF834VWT | ENCFF190RZM | ENCFF036KBQ | ENCFF110AUF | ENCFF289DWP | ENCFF574PIN | ENCFF635XBE |
| H3K36me3 | ENCFF431XVV | ENCFF345QSP | ENCFF247PKZ | ENCFF942SCO | ENCFF863VMA | ENCFF532OYN | ENCFF259MAY |
| H3K9me3 | ENCFF542CZT | ENCFF701GHA | ENCFF071KRK | ENCFF710FWU | ENCFF426TFY | ENCFF059AXW | ENCFF409PTA |

*Note*: The contents of the table are the accession number of the corresponding data in the ENCODE database.
