# Supplementary material for: COCOA: A Framework for Fine-scale Mapping of Cell-type-specific Chromatin Compartments Using Epigenomic Information
Source: Genomics Proteomics Bioinformatics. 2024 Dec 26;22(6):qzae091. doi: 10.1093/gpbjnl/qzae091 (PMC11993304; doi:10.1093/gpbjnl/qzae091)
Supplement: qzae091_Supplementary_Data [file qzae091_supplementary_data.zip › qzae091_Supplementary_Data/Table S3.docx]

**Table S3 Summary table (performance evaluation)**

| **Chr** | **MAE** | **SSIM** | **PSNR** |
| --- | --- | --- | --- |
| 2 | 0.2318 | 0.3500 | 11.94 |
| 4 | 0.2667 | 0.3086 | 10.81 |
| 6 | 0.1731 | 0.4220 | 13.84 |
| 8 | 0.2216 | 0.4261 | 12.19 |
| 10 | 0.1971 | 0.4321 | 12.76 |
| 12 | 0.1916 | 0.4308 | 13.18 |
| 14 | 0.2104 | 0.4394 | 12.16 |
| 16 | 0.1601 | 0.4352 | 13.82 |
