# Supplementary material for: COCOA: A Framework for Fine-scale Mapping of Cell-type-specific Chromatin Compartments Using Epigenomic Information
Source: Genomics Proteomics Bioinformatics. 2024 Dec 26;22(6):qzae091. doi: 10.1093/gpbjnl/qzae091 (PMC11993304; doi:10.1093/gpbjnl/qzae091)
Supplement: qzae091_Supplementary_Data [file qzae091_supplementary_data.zip › qzae091_Supplementary_Data/Table S4.docx]

**Table S4 Summary table (multiple resolution)**

| **Chr** | **MAE** | **MSE** | **SSIM** | **PSNR** |
| --- | --- | --- | --- | --- |
| 16 (10k) | 0.2404 | 0.0758 | 0.1750 | 11.20 |
| 17 (10k) | 0.2051 | 0.0565 | 0.1961 | 12.48 |
| 18 (10k) | 0.2169 | 0.0614 | 0.1828 | 12.12 |
| 16 (1k) | 0. 0603 | 0.0123 | - | 19.10 |
| 17 (1k) | 0.1389 | 0.0273 | - | 15.64 |
| 18 (1k) | 0.1634 | 0.0346 | - | 14.61 |

*Note*: “-” indicates metrics that are not available due to computational resource constraints.
