# Supplementary material for: COCOA: A Framework for Fine-scale Mapping of Cell-type-specific Chromatin Compartments Using Epigenomic Information
Source: Genomics Proteomics Bioinformatics. 2024 Dec 26;22(6):qzae091. doi: 10.1093/gpbjnl/qzae091 (PMC11993304; doi:10.1093/gpbjnl/qzae091)
Supplement: qzae091_Supplementary_Data [file qzae091_supplementary_data.zip › qzae091_Supplementary_Data/Table S5.docx]

**Table S5 Summary table (cell-type-specific prediction)**

| **Chr** | **MSE** | **MAE** | **SSIM** | **PSNR** |
| --- | --- | --- | --- | --- |
| 12 | 0.0620 | 0.2034 | 0.3566 | 12.08 |
| 13 | 0.0871 | 0.2615 | 0.2690 | 10.60 |
| 14 | 0.1212 | 0.3000 | 0.3200 | 9.16 |
| 15 | 0.0836 | 0.2466 | 0.3362 | 10.78 |
| 16 | 0.0804 | 0.2342 | 0.3208 | 10.95 |
| 17 | 0.0667 | 0.2125 | 0.3981 | 11.74 |
| 18 | 0.0786 | 0.2376 | 0.2564 | 11.04 |
| 19 | 0.0585 | 0.1953 | 0.4462 | 12.33 |
