# Supplementary figures and images for: COCOA: A Framework for Fine-scale Mapping of Cell-type-specific Chromatin Compartments Using Epigenomic Information
Source: Genomics Proteomics Bioinformatics. 2024 Dec 26;22(6):qzae091. doi: 10.1093/gpbjnl/qzae091 (PMC11993304; doi:10.1093/gpbjnl/qzae091)

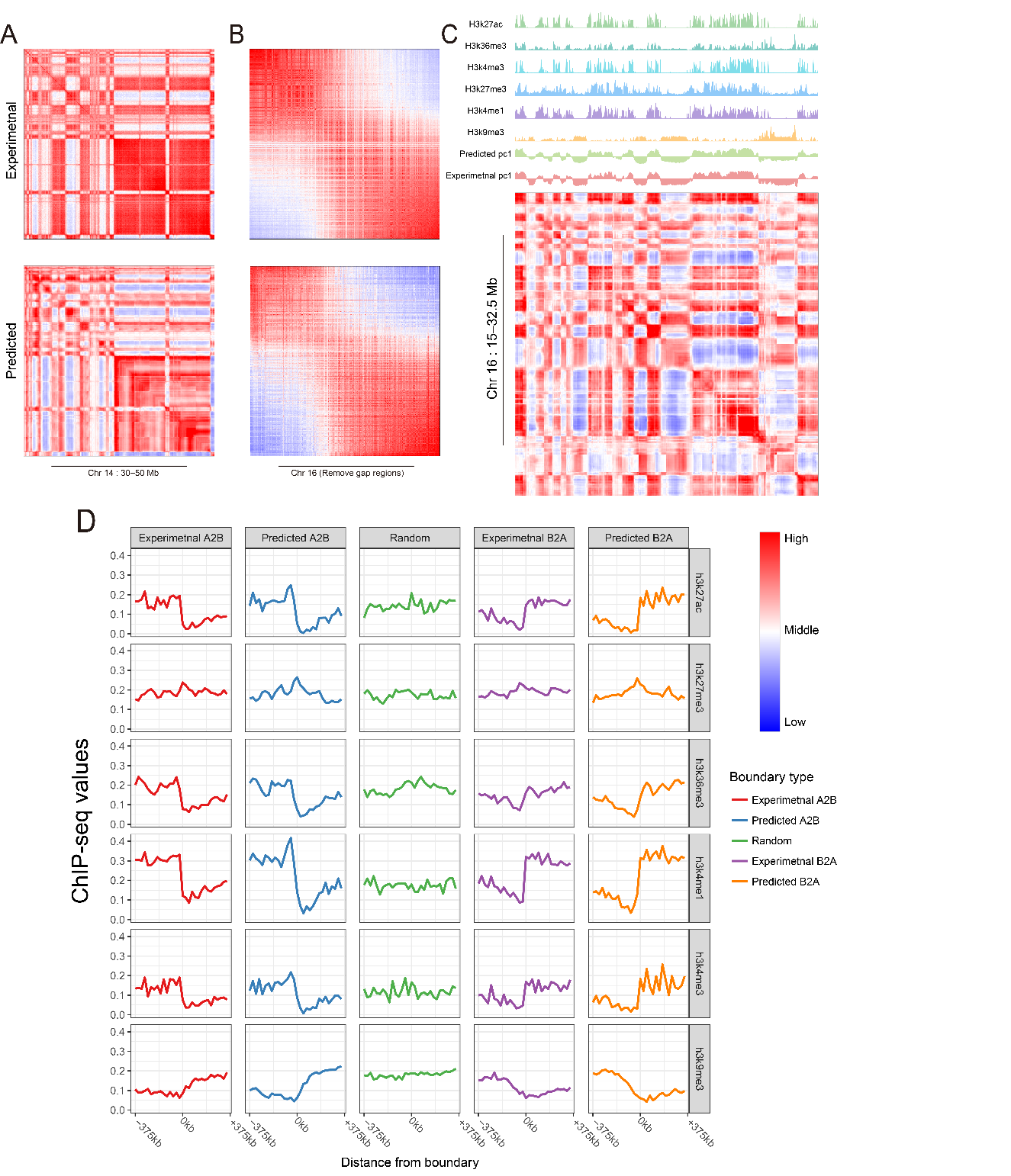

Supplement: qzae091_Supplementary_Data [file qzae091_supplementary_data.zip › qzae091_Supplementary_Data/Figure S1.docx]

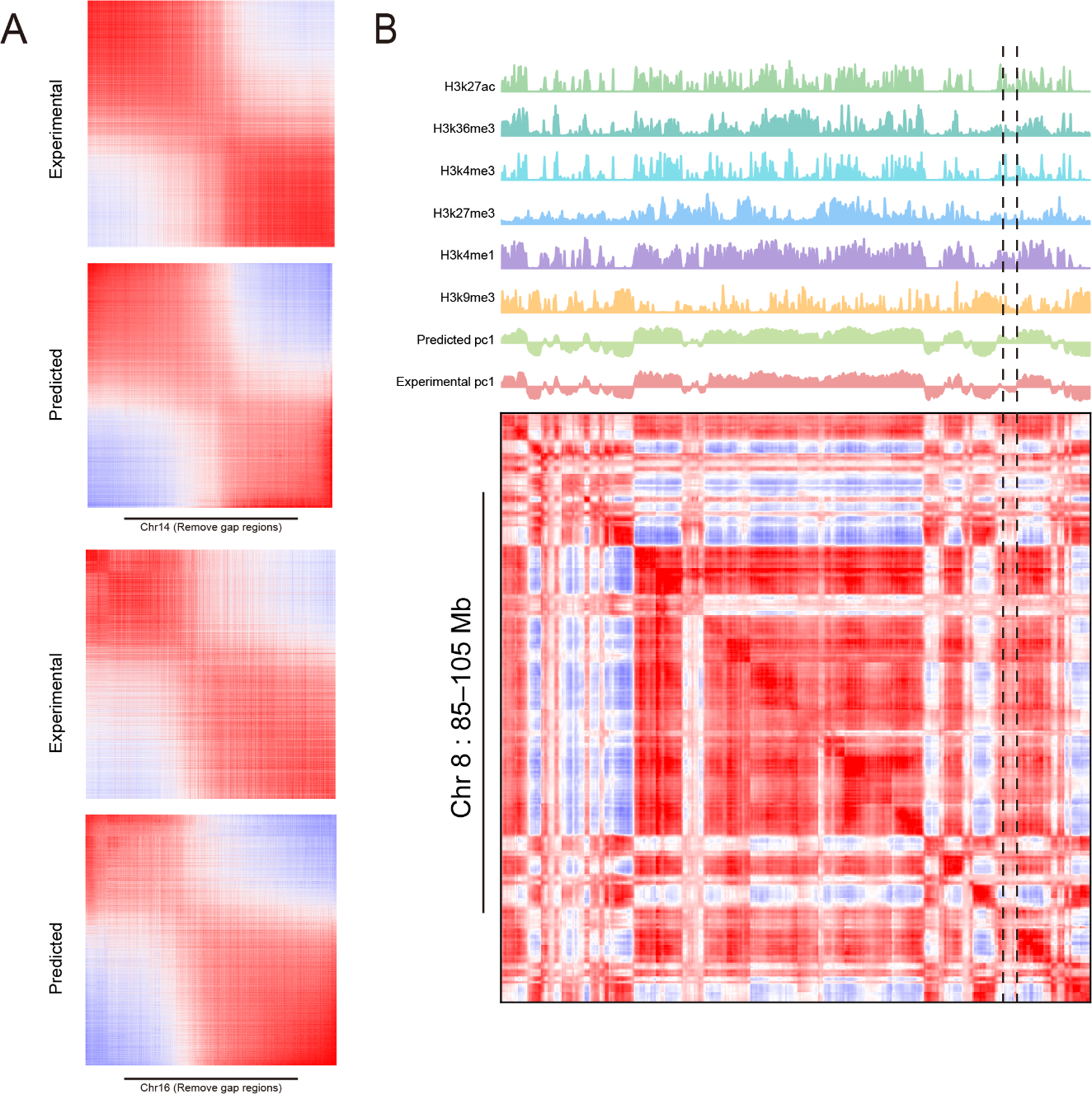

Supplement: qzae091_Supplementary_Data [file qzae091_supplementary_data.zip › qzae091_Supplementary_Data/Figure S2.docx]

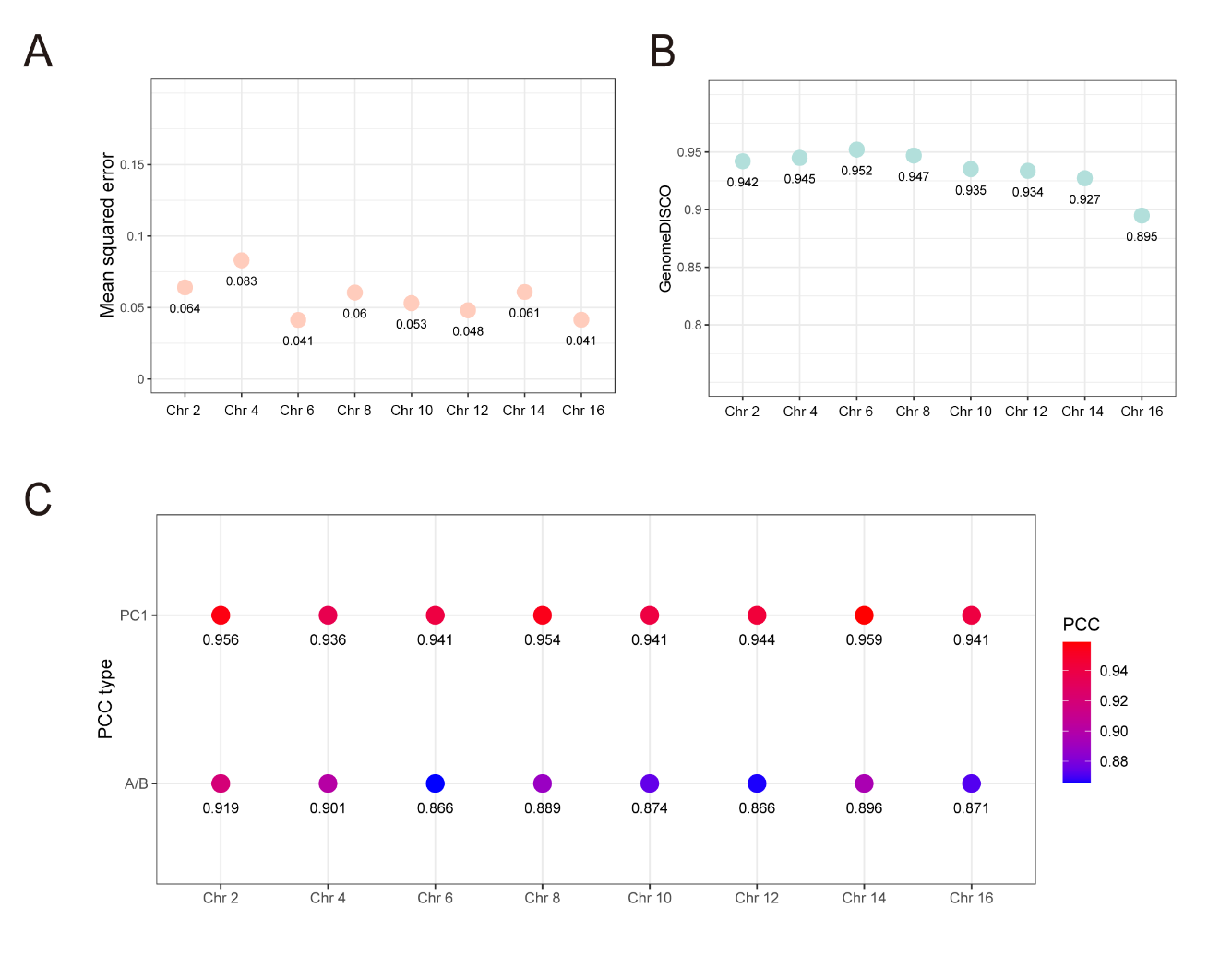

Supplement: qzae091_Supplementary_Data [file qzae091_supplementary_data.zip › qzae091_Supplementary_Data/Figure S3.docx]

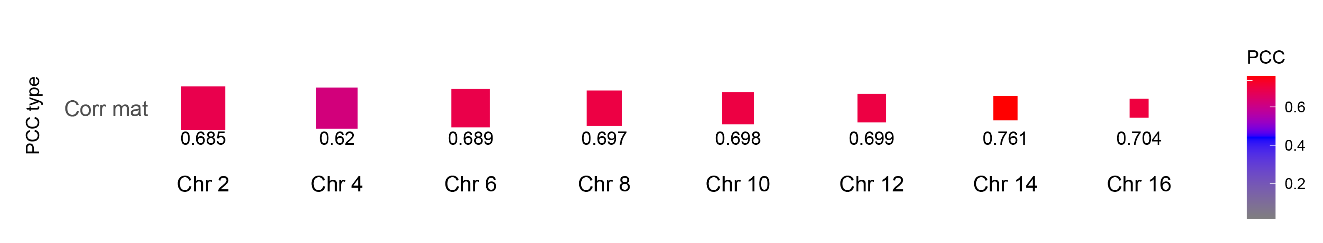

Supplement: qzae091_Supplementary_Data [file qzae091_supplementary_data.zip › qzae091_Supplementary_Data/Figure S4.docx]

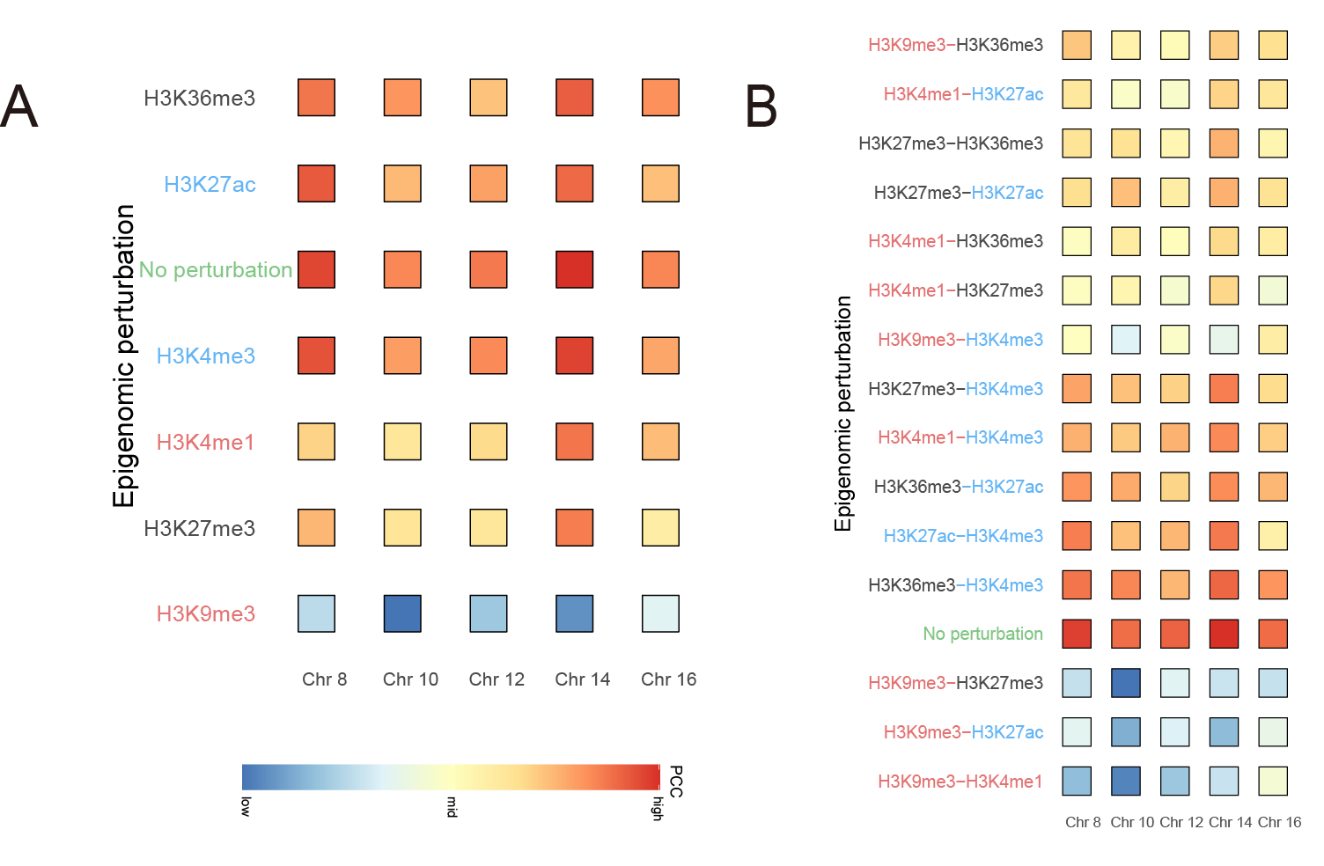

Supplement: qzae091_Supplementary_Data [file qzae091_supplementary_data.zip › qzae091_Supplementary_Data/Figure S5.docx]

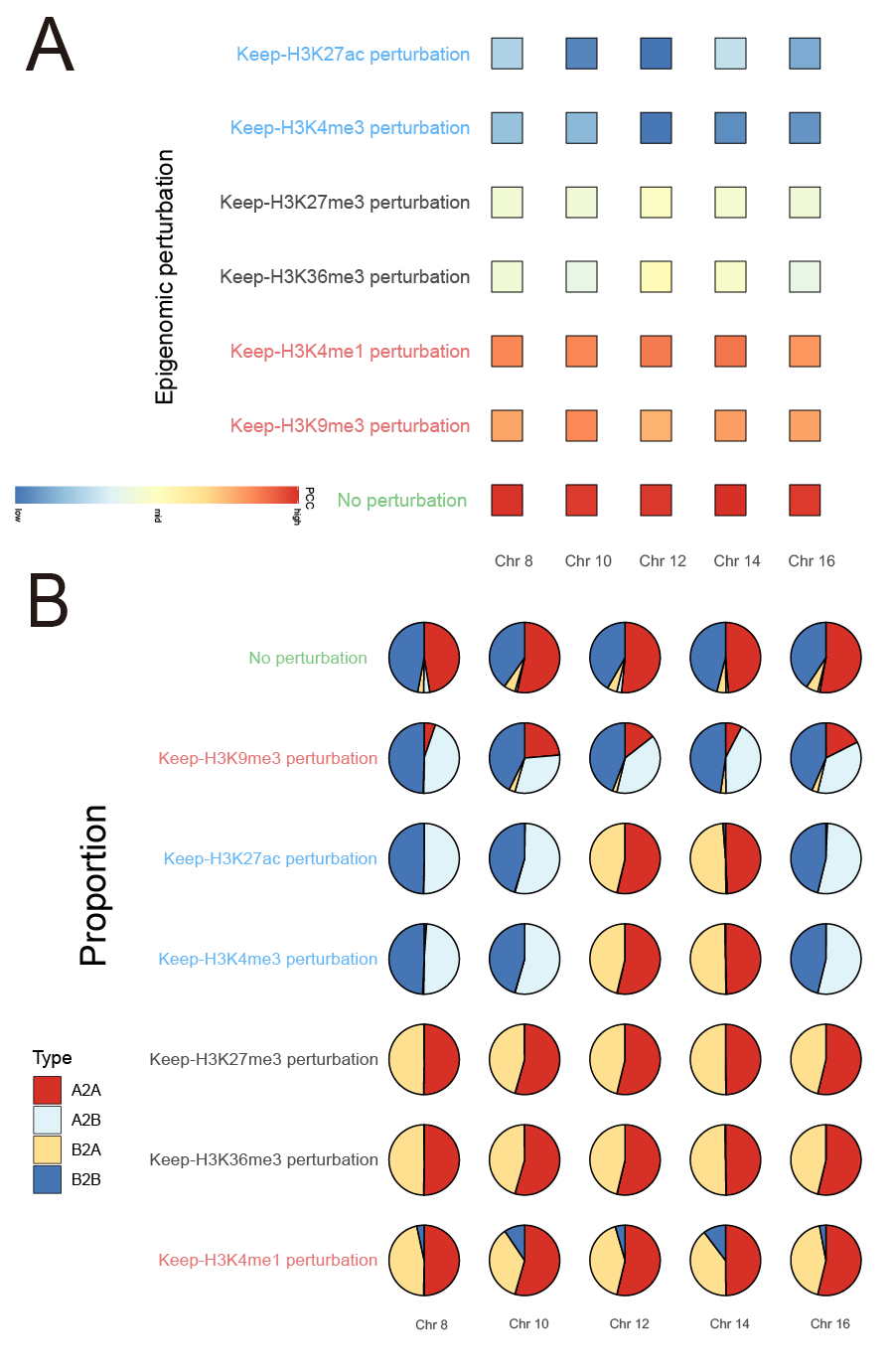

Supplement: qzae091_Supplementary_Data [file qzae091_supplementary_data.zip › qzae091_Supplementary_Data/Figure S6.docx]

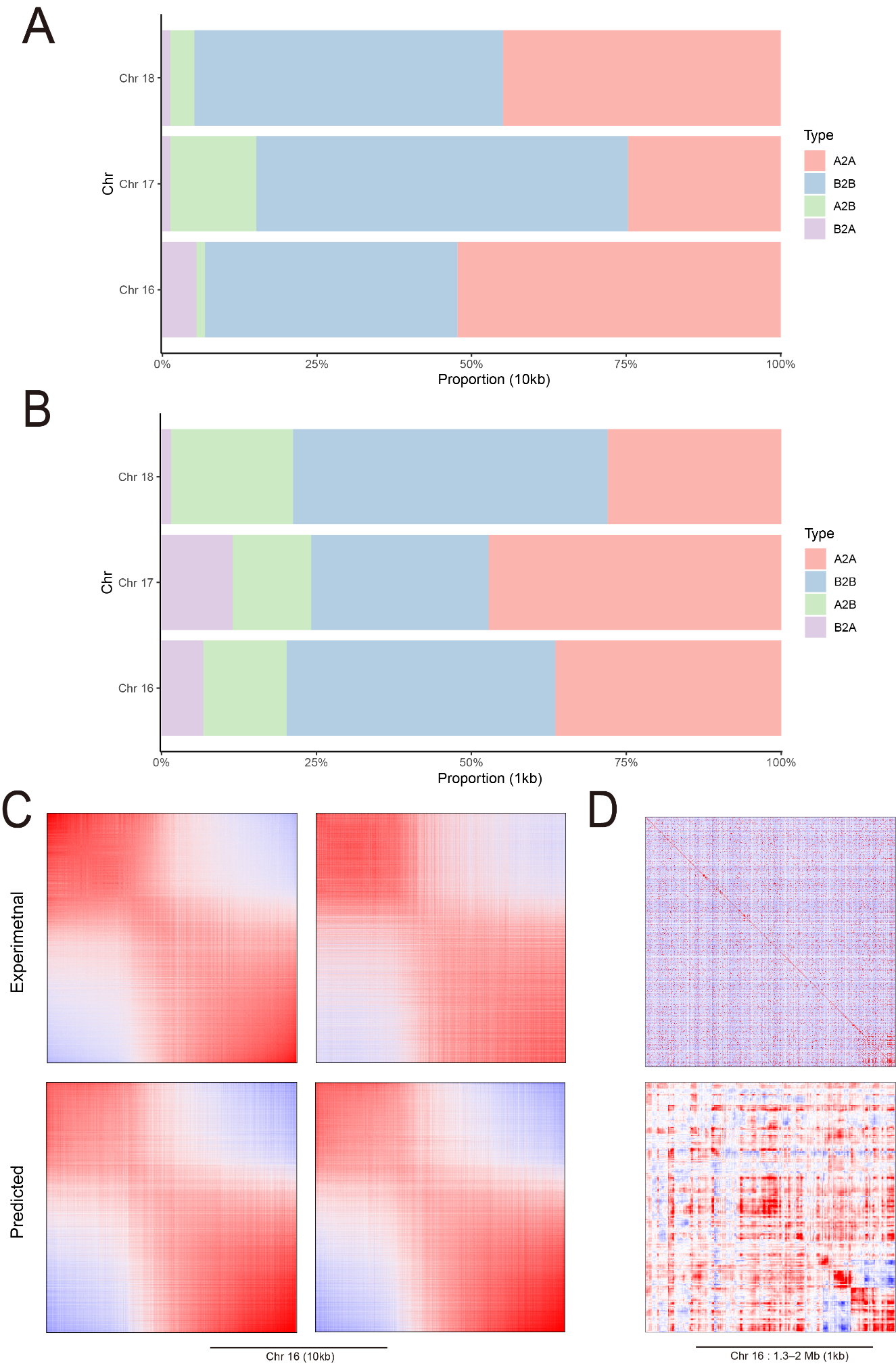

Supplement: qzae091_Supplementary_Data [file qzae091_supplementary_data.zip › qzae091_Supplementary_Data/Figure S7.docx]

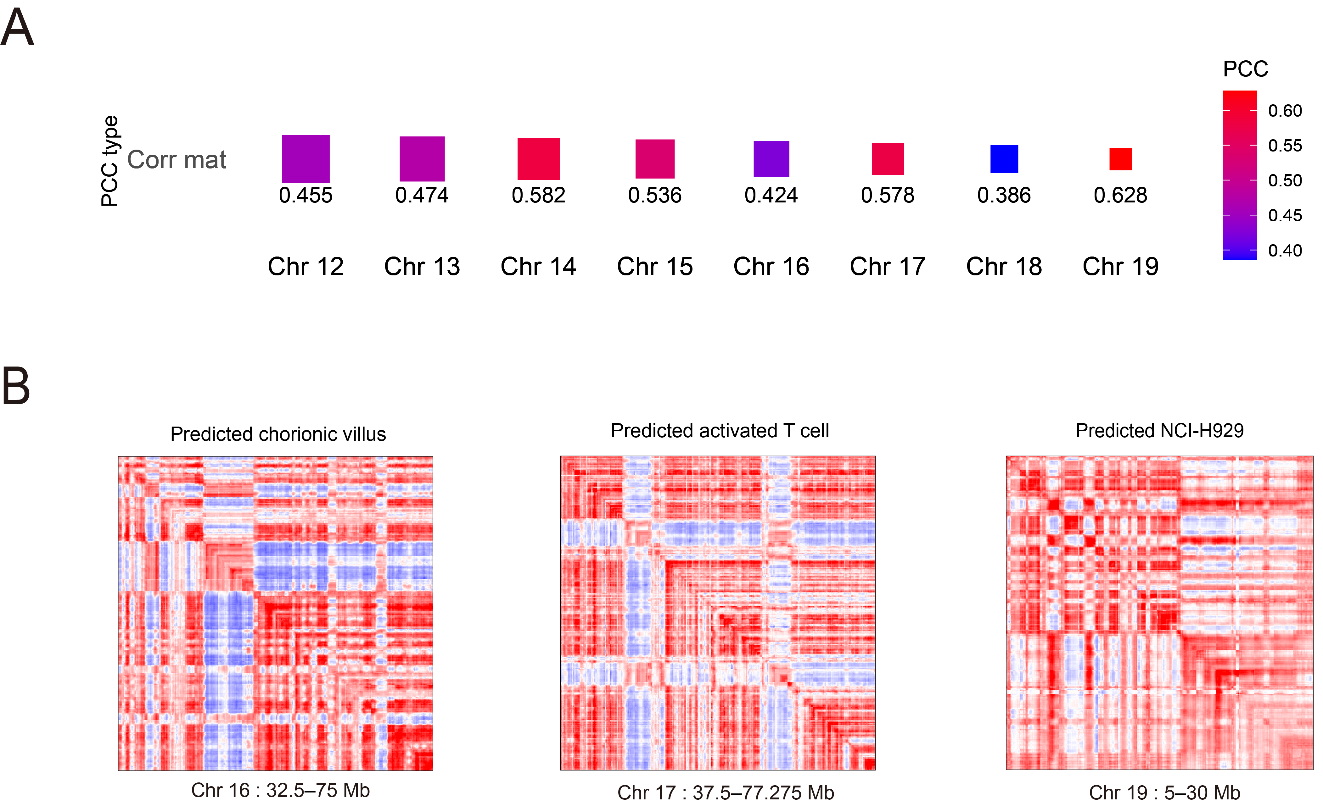

Supplement: qzae091_Supplementary_Data [file qzae091_supplementary_data.zip › qzae091_Supplementary_Data/Figure S8.docx]
